# Raloxifene as Treatment for Various Types of Brain Injuries and Neurodegenerative Diseases: A Good Start †

**DOI:** 10.3390/ijms21207586

**Published:** 2020-10-14

**Authors:** Leo Veenman

**Affiliations:** Israel Institute of Technology, Faculty of Medicine, Rappaport Institute of Medical Research, 1 Efron Street, P.O. Box 9697, Haifa 31096, Israel; veenmanl@techunix.technion.ac.il or jehudaveenman@gmail.com

**Keywords:** Alzheimer, Parkinson, stroke, traumatic brain injury (TBI), neurodegeneration, Raloxifene, selective estrogen receptor modulator(s), FDA approved, gene expression, mitochondrial activity, cure

## Abstract

Recent studies have shown that the selective estrogen receptor modulator (SERM) raloxifene had pronounced protective effects against progressing brain damage after traumatic brain injury (TBI) in mice. These studies, indicating beneficial effects of raloxifene for brain health, prompted the study of the history and present state of knowledge of this topic. It appears that, apart from raloxifene, to date, four nonrelated compounds have shown comparable beneficial effects—fucoidan, pifithrin, SMM-189 (5-dihydroxy-phenyl]-phenyl-methanone), and translocator protein (TSPO) ligands. Raloxifene, however, is ahead of the field, as for more than two decades it has been used in medical practice for various chronic ailments in humans. Thus, apart from different types of animal and cell culture studies, it has also been assessed in various human clinical trials, including assaying its effects on mild cognitive impairments. Regarding cell types, raloxifene protects neurons from cell death, prevents glial activation, ameliorates myelin damage, and maintains health of endothelial cells. At whole central nervous system (CNS) levels, raloxifene ameliorated mild cognitive impairments, as seen in clinical trials, and showed beneficial effects in animal models of Parkinson’s disease. Moreover, with stroke and TBI in animal models, raloxifene showed curative effects. Furthermore, raloxifene showed healing effects regarding multiple sclerosis (MS) and amyotrophic lateral sclerosis (ALS) in cell culture. The adverse biological signals typical of these conditions relate to neuronal activity, neurotransmitters and their receptors, plasticity, inflammation, oxidative stress, nitric oxide, calcium homeostasis, cell death, behavioral impairments, etc. Raloxifene favorably modulates these signals toward cell health—on the one hand, by modulating gene expression of the relevant proteins, for example by way of its binding to the cell nuclear estrogen receptors ERα and ERβ (genomic effects) and, on the other hand (nongenomic effects) by modulation of mitochondrial activity, reduction of oxidative stress and programmed cell death, maintaining metabolic balance, degradation of Abeta, and modulation of intracellular cholesterol levels. More specifically regarding Alzheimer’s disease, raloxifene may not cure diagnosed Alzheimer’s disease. However, the onset of Alzheimer’s disease may be delayed or arrested by raloxifene’s capability to attenuate mild cognitive impairment. Mild cognitive impairment is a condition that may precede diagnosis of Alzheimer’s disease. In this review, relatively new insights are addressed regarding the notion that Alzheimer’s disease can be caused by bacterial (as well as viral) infections, together with the most recent findings that raloxifene can counteract infections of at least some bacterial and viral strains. Thus, here, an overview of potential treatments of neurodegenerative disease by raloxifene is presented, and attention is paid to subcellular molecular biological pathways that may be involved.

## 1. Introduction

**Figure 1:**
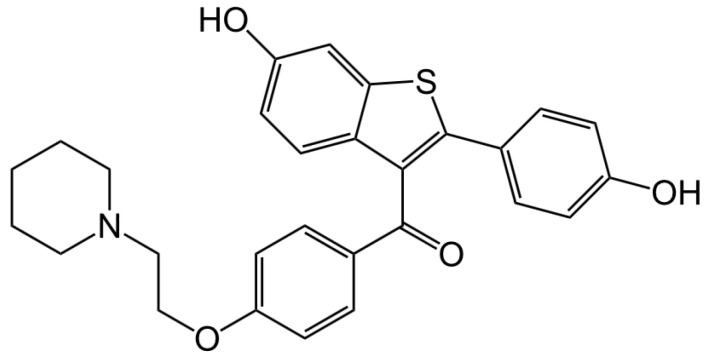
Chemical structure of raloxifene. By Jü - own work, CC0, available online: https://commons.wikimedia.org/w/index.php?curid=16071989. Raloxifene hydrochloride, C_28_H_27_NO_4_S•HCl, has a molecular weight of 510.05 g/mol. IUPAC name: [6-hydroxy-2-(4-hydroxyphenyl)-1-benzothiophen-3-yl]-[4-(2-piperidin-1-ylethoxy)phenyl]methanone;hydrochloride.

This review focuses on raloxifene (Figure 1), a selective estrogen receptor modulator (SERM).

This review compiles data on raloxifene and other selective estrogen receptor modulators (SERMs) relevant for their promising application as treatments of brain injury and disease. In a recent article, Honig et al. [1] suggested that raloxifene (Figure 1) actually may be ready for a clinical trial, Phase 2, for testing its efficacy to treat mild traumatic brain injury (TBI). Importantly, it has been used as medication for various (very serious) diseases for over 20 years (i.e., it is FDA approved, and well established as safe). Raloxifene was first developed by Eli Lilly and marketed as Evista, which was approved by the FDA to treat postmenopausal osteoporosis more than twenty years ago. Since then, it has also been FDA-approved to reduce the risk of invasive breast cancer in postmenopausal women, and is currently available in generic form. Furthermore, raloxifene acts as an estrogen agonist on bone and lipid metabolism and as an estrogen antagonist for reproductive tissues [2,3]. Animal studies suggest that raloxifene may affect brain function as well, although the effects of raloxifene on the human brain remain to be established in more detail [4,5,6]. 

Early on in the use of raloxifene, it was shown that after 12 months of treatment of postmenopausal women with raloxifene (60 and 120 mg/day), the application of performance testing with the Memory Assessment Clinics (MAC) battery, the Walter Reed Performance Assessment Battery (PAB), and the Geriatric Depression Scale (GDS), did not show negative effects of raloxifene [4]. Raloxifene also has no evident adverse hormonal side effects, and is safe for use in men, as seen in clinical trials to treat bone fracture [7], prostate cancer [8], and acromegaly [9]. In male rats, raloxifene was found to be a pure estrogen antagonist and a physiological antagonist of androgen action [10]. Apart from these examples of peer-reviewed research, information provided by Lilly, the company from where raloxifene originated, states that raloxifene provided in daily doses from 30 to 600 mg/kg, is well tolerated. Nonetheless, contraindications provided by the FDA (https://www.accessdata.fda.gov/drugsatfda_docs/label/2007/022042lbl.pdf) have to be kept in mind. These contraindications include preceding extant blood circulatory problems, for example, active or past history of venous thromboembolism, including deep vein thrombosis, pulmonary embolism, and retinal vein thrombosis, as well as documented coronary heart disease or increased risk for major coronary events. Regarding distribution in the body, measuring the relative levels of raloxifene in rat tissues, after giving a simple oral dose, the highest levels of raloxifene were observed in the liver, lung, spleen, followed by the heart and kidney [11]. The lowest level was found in the brain. This study also indicated that raloxifene distributes rapidly and moderately into tissues, such as the liver, lung, and spleen [11].

During the time that raloxifene became used as a treatment for osteoporosis, it was already known that estrogen can improve cognition and delays the onset of Alzheimer’s dementia (although it cannot cure diagnosed Alzheimer’s disease), and favorably influences the feeling of well-being [12]. However, estrogens can have unfavorable side effects in tissues and organs outside the CNS. Nonetheless, it was predicted that selective estrogen receptor modulators (SERMs) at one point would lead to clinical trials of CNS-targeted SERMs [12]. Indeed, a variety of SERMs were developed to retain the favorable effects of estrogen, while minimizing their adverse side effects when used for menopausal treatments. Moreover, the neuroprotective effects of two specific SERMs, raloxifene and arzoxifene, became known at this time [13]. 

Subsequently, it was found that raloxifene and other SERMs favorably affect the nervous system [14]. In particular, in various experimental models of neural trauma, brain inflammation, and neurodegenerative diseases, application of SERMs provided neuroprotective mechanisms, as well as reductions in neural damage. This was associated with the capabilities of these SERMs to ameliorate cognitive impairments and affective disorders [14,15].

Leading possibilities, whereby SERMs might provide neuroprotection, were thought to include: (1) acting via the genomic estrogen receptors (ERα and ERβ) on functional pathways to modulate gene transcription; (2) acting via nongenomic pathways, including activation of cell signalers, such as mitogen-activated protein kinases and/or phosphatidylinositol-3-kinase /protein kinase B; (3) direct antioxidant free-radical scavenging, i.e., bypassing receptors, which is primarily attained with pharmacological doses of estrogen; (4) acting via other potential mediatory factors, such as growth factors; and (5) modulating the functions of glial cells in general, as well modulating neuronal activities directly [15]. Since then, these mentioned functional pathways, and various other entities, mechanisms, pathways, and systems, have been studied to better understand how SERMs can exert their beneficial effects on brain injury and brain disease, as discussed in this review.

## 2. Raloxifene in the Context of Other Advances in Treatments of Neurodegeneration

Raloxifene is not the only agent that shows promise in the search for development for treatment of neurodegeneration. In a search targeting research published during the year 2019 (keywords: Mice, TBI, treatment), 167 studies came up from PubMed (https://pubmed.ncbi.nlm.nih.gov/). In these 167 studies, as many compounds had been assayed for efficaciousness on TBI in mice, a handful showed effectiveness of more than 90% curation, according to the measurements presented by these studies themselves. Raloxifene is one of these quite efficacious compounds tested for mouse TBI [1]. Other agents that showed the same levels of success were hyperbaric oxygen treatment, pifithrin, SMM-189, translocator protein (TSPO) ligands, and fucoidan. Other compounds also showed various levels of success, usually around 50% of effectiveness compared to their controls. Raloxifene stood out for various reasons. First of all, because, at least in mice, first application of raloxifene can be as late as 48 h after induction of TBI, and then it still provides close to 100% curation [1]. This may be true for other compounds, too, but such late time points are typically not tested, or do not provide curation. Note, standing dogma is that treatment should start within a handful of hours (or less) after TBI induction, and cannot be effective if started at later time points (e.g., [16,17]). For a healthcare situation, this 48 h time point after TBI possibility demonstrated for raloxifene by Honig et al. [1] implies that a victim/patient can be brought to the clinic without undue time pressure. The other, indeed most interesting, aspect was that raloxifene is an FDA approved medicine that has been in use for various treatments for more than 20 years. Furthermore, its promising effects on cognition have been part of clinical trials. This review aims to elucidate the aspects of raloxifene researched in relation to brain damage due to injury and disease. 

The other efficacious agents regarding TBI hinted at above are (very) summarily touched upon here. As for raloxifene, the criteria for their selection were: more than 90% curation and applicability at least 4 h after induction of TBI and later. Of these agents meeting these criteria, only one treatment has reached clinical application for treatment of TBI (namely, hyperbaric oxygen treatment) [18,19,20]. Another very efficacious agent, at least for mouse TBI that has been studied in cell culture and animal models for almost 20 years now, is pifithrin [21,22]. Moreover, compounds acting on the cannabinoid type-2 (CB-2) receptor (including SMM-189, as well as raloxifene) can be very effective for treatment of TBI [1,23,24,25,26]. Finally, two other promising representatives of these five efficacious agent are ligands for the mitochondrial 18-kDa translocator protein (TSPO) and a brown seaweed product fucoidan, reviewed elsewhere [27,28,29]. By compiling data from raloxifene studies, this review also hopes to provide aid and direction for attempts to establish standards for evaluation of drugs that present themselves as candidates for treatment of brain damage due to injury and disease. Fortunately, raloxifene not only appears efficacious, but the more than two decennia of raloxifene research also covers a wide spectrum of parameters—from molecular biological mechanism to clinical trials—which, indeed, are most relevant for gaining insight into the mechanisms associated with neurodegeneration as part of the chronic processes of brain damage due to injury and disease.

## 3. Brain Cell Types and the Effects of Raloxifene

**Figure 2:**
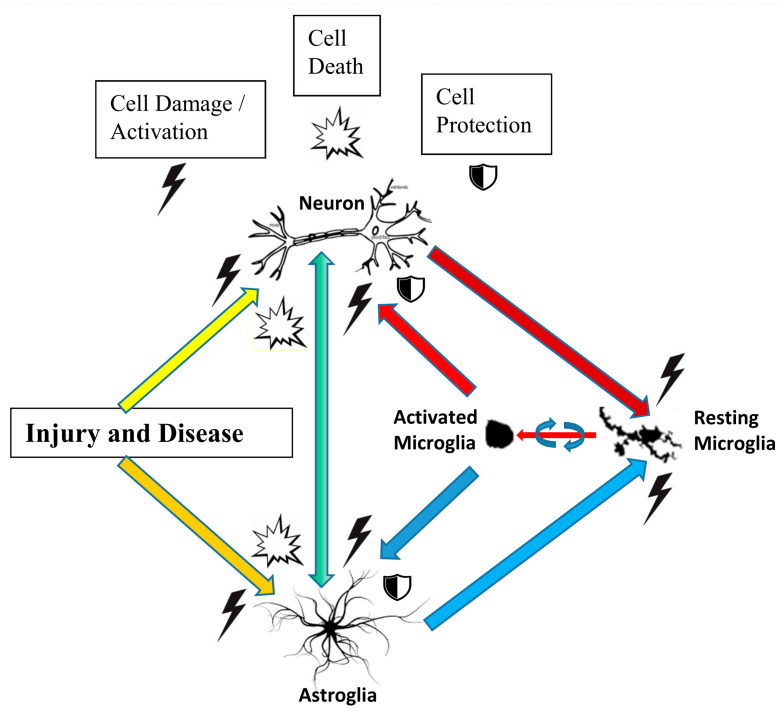
Schematic overview of interactions between neurons, astroglia, and microglia in the CNS, as they can occur due to brain injury and brain disease. The effects of raloxifene and other selective estrogen receptor modulators (SERMs) on these cell types and their interactions in relation to brain injury and disease are discussed in Section 3: Brain Cell Types and the Effects of Raloxifene.

The brain contains various cell types (neurons, astrocytes, microglia, oligodendrocytes, endothelial cells), which are discussed in this Section 3, in relation to raloxifene application. It is also well known that these various cell types interact in intricate ways, in relation to brain disease and brain injury, either reducing the damage or contributing to the damage (Figure 2). Briefly, progressing brain damage due to injury and disease typically includes damage and death of neurons and astrocytes, which, in turn causes microglia activation [27,28,29,30,31,32]. Briefly, by its mechanical force alone, an impact on the head or other injury can induce neuronal and astrocytic damage and death. If, and when disease is the damaging event, induction of microglial activation may be the first apparent phenomenon. Subsequently, neuronal and astrocytic death, damage, and activation can cause more microglial activation, and vice versa. Acute microglial activation may help to maintain neuronal and astrocytic health. In contrast, chronic microglial activity may cause further damage and even death to other neural cells in the brain. These processes and events involving neurons, astrocytes, and microglia are presented schematically in Figure 2 at the end of this Section 3.

### 3.1. Neurons

To mention very briefly, neurons present the fundamental functional units of nervous tissue assembling circuitry for receiving and transmitting impulses. Morphologically, neurons display two types of highly differentiated cytoplasmic processes (neurites), one type being dendrites, and the other type being axons. From just one to a large number of dendrites can protrude from a neuron’s cell body. Dendrites serve for reception of input, typically from other neurons. Axons project away from the cell body to conduct impulses to other neurons or to target organs. Neurons typically present solitary axons, which can branch close or distant from the cell body. Regarding this review, ovarian hormones can affect the physiological activities of neurons, including their signal transducing dendrites and axons. For example, prior to the marketing of raloxifene, it was shown that the antiestrogen keoxifene modulated the ontogenetic processes of myelination and the differentiation of neurons in telencephalic song motor centers, as well as cerebellar structures, associated with the seasonal behavior of male Zebra finches [33]. Note: associated with seasonal song, song motor centers in the brains of songbirds can expand and decrease in volume, including proliferation of neurons and growth of dendrites and axons. 

After these observations in birds, it was also seen in the brains of mammals that ovarian hormones were able to regulate neuronal development and morphology, and neuronal activity in association with behavioral performance. For example, ovarian hormones were found to modulate dopamine neuron firing activity in the ventral tegmental area (VTA) induced by acute cocaine in intact female and ovariectomized rats [34]. In particular, estrogen synergized with the inhibitory effect of cocaine on VTA dopamine neuron activity [34]. Moreover, pretreatment with the estrogen receptor antagonist raloxifene, or the selective estrogen α (ERα) receptor antagonist Y134, attenuated the cocaine-inhibition of DA neuron firing rates [34]. Neuroprotective effects of estradiol have been well characterized in various animal experimental models, as was discussed by Ciriza et al. [35]. In their own experiments [35], they assayed the effects of the SERMs raloxifene, tamoxifen, lasofoxifene (CP-336,156), and bazedoxifene (TSE-424), as well as 17β-estradiol in the hippocampus after the administration of the excitotoxin kainic acid to adult ovariectomized rats. This application of kainic acid injection induced a significant neuronal loss in the hilus of the hippocampus. Estradiol, raloxifene (0.4–2 mg/kg), tamoxifen (0.4–2 mg/kg), and bazedoxifene (2 mg/kg) prevented this neuronal loss [35]. These and other studies demonstrated the neuroprotective and anti-inflammatory properties of raloxifene and tamoxifen in several experimental models of neurodegeneration [36]. Furthermore, raloxifene and tamoxifen counteracted cognitive deficits caused by gonadal hormone deprivation in male rats. In this context, raloxifene and tamoxifen can regulate the number and geometry of dendritic spines on CA1 pyramidal neurons of the rat hippocampus and of spines on apical dendrites of pyramidal neurons in the prefrontal cortex [36,37]. In some detail, ovariectomized rats treated with estradiol benzoate, tamoxifen, or raloxifene showed significant increases in the numerical density of spines on secondary apical dendrites of layer III pyramidal neurons in the prelimbic/infralimbic prefrontal cortex, together with a better performance in the Y maze [37]. Furthermore, SERMs reduced inflammatory response of glial cells in this paradigm, in addition to modulation of synaptic plasticity in the hippocampus, accompanying reduction in anxiety and depression, and improvement of cognition [37]. Thus, summarizing these papers here shows that raloxifene and other SERMs affect neuronal activities at various levels of organization, ranging from physiological activity, via morphological changes and neurodevelopment, to cell protection, altogether associated with mental processes and behavior, and their protection. This has implications on our understanding of neurodegeneration, as it can occur with brain disease and brain injury—such as Alzheimer’s disease, Parkinson’s disease, stroke, brain penetrating wounds, and blunt traumatic brain injury—which are the subjects of Section 5. 

Neuronal activity related to memory and learning: in rodents as well as humans, cognitive tasks are impaired by androgen deprivation [38]. Androgen replacement can reverse these deficits. It is thought that the effects of androgens in the male brain may be mediated in part by metabolism of androgens to estradiol or 3-α androstanediol, and the consequent activation of estrogen receptors [38]. Briefly, to gain information in this respect, it was assessed whether the administration of estradiol benzoate, the estrogen receptor α (ERα) selective agonist propyl-pyrazole-triol (PPT), or the estrogen receptor β (ERβ) selective agonist diarylpropionitrile (DPN), can affect performance of androgen-deprived male Wistar rats in a cross-maze test [38]. In addition, the effects of the SERMs raloxifene and tamoxifen were tested in this paradigm. The behavior of the rats was assessed 2 weeks after orchidectomy or sham surgery. As expected, orchidectomy impaired acquisition in the cross-maze test. Giving estradiol benzoate or the selective estrogen receptor β agonist DPN to orchidectomized animals significantly improved acquisition in the cross-maze test compared to vehicle control [38]. Moreover, raloxifene and tamoxifen, given at a dose of 1 mg/kg, improved acquisition by orchidectomized animals, while doses of 0.5 or 2 mg/kg did not have this effect. These findings suggest that estrogenic compounds with affinity for ERβ, and, thus, raloxifene and tamoxifen may enhance cognitive performance in androgen-deprived males. This may hopefully have implications for persons with mild cognitive impairment [5,39,40,41], as discussed with the subject of Alzheimer’s disease, Dementia, in Section 5. 

In one study, it was shown that estradiol regulates the expression of hippocampal parvalbumin in hippocampal interneurons as well as hippocampus-dependent spatial memory in mice [42]. Parvalbumin interneurons generate neuronal oscillatory activity in the gamma frequency range (30–80 Hz) (gamma-band oscillations) that is considered associated with higher cognitive functions. To study modulations by estradiol and raloxifene of hippocampal gamma-band oscillations during spatial memory performance, a subcutaneous pellet of estradiol or raloxifene, or placebo was implanted in prepubescent ovariectomized mice [42]. Then, during adulthood, while performing a Y-maze hippocampus-dependent spatial memory task, local field potentials were recorded in their dorsal hippocampus [42]. First of all, ovariectomy caused deficits in spatial memory, accompanied by a significant reduction in hippocampal gamma-band oscillations, specifically during decision making. Estradiol and raloxifene provided treatment for such behavioral and electrophysiological deficits [42]. This suggests potential beneficial effects of raloxifene for treatments of neurobehavioral deficits.

Gap junctions: gap junctions are regulated channels traversing the plasma membranes of two adjacent cells, allowing direct passage of various molecules, ions, and electrical impulses from one cell to another [43,44]. In the nervous system, gap junctions allow for rapid, typically bi-directional, transmission of signals from cell to cell. In this way, a network of multicellular communication is established. One of the general functions of gap junctions is taking part in neuronal differentiation and survival [45]. Potential neural effects of raloxifene and tamoxifen on the effects of gap junctions related to differentiation were studied in the human teratocarcinoma cell line NTera2/D1 [45]. The mentioned study targeted retinoic acid-dependent neuronal differentiation regulated by gap junctions formed of connexin43. Applying 10 µmol/L tamoxifen and raloxifene to NTera2/D1 cells during weeks 1 and 2 of a 6-week retinoic acid-driven differentiation schedule impaired the neuronal differentiation of these cells otherwise induced during this schedule. However, such treatment during weeks 5 and 6 of this schedule did not affect this differentiation [45]. Thus, modulation by raloxifene and tamoxifen of the gap junctions connecting NTera2/D1 cells appears to affect early neuronal differentiation, but does not affect differentiated, mature neurons. In comparison to the study of [46] reviewed in Section 5.4, Cerebral Ischemia, this may suggest that SERMs can modulate neuronal systems in process of development or regeneration after damage, but when fully differentiated, neuronal systems will not suffer aberrations from their mature, stabilized condition when exposed to SERMs. This may reflect homeostatic functions of SERMs to serve the return of aberrant states back to normalcy, while maintaining normal, healthy states of neuronal systems.

Calcium: calcium dyshomeostasis is one of the causes for memory impairment. For example, intracellular Ca^2+^ dyshomeostasis appears to underlie cognitive deficits seen in normal aging as well as degenerative neurologic diseases [47]. Thus, it is interesting to know whether raloxifene can neutralize the adverse effects of glutamate on cultured neurons by regulation of calcium oscillations [48]. It was investigated whether raloxifene could affect the glutamate-induced Ca^2+^ overload in rat cultured cortical neurons [49]. With whole-cell recording, the effects of raloxifene on N-methyl-D-aspartate (NMDA)-evoked and voltage-activated Ca^2+^ currents in cultured cortical neurons were measured. Pre-exposure of cortical neurons to raloxifene (0.5 µM–10 µM) for 3 min attenuated intracellular Ca^2+^ increase that is otherwise induced by glutamate (300 µM) for 1 min. The action of raloxifene was reversible after washout of the neurons in cell culture [49]. The estrogen receptor antagonist ICI 182,780 (**ICI**, fulvestrant, Faslodex), and the endoplasmic reticulum Ca^2+^-ATPases’ inhibitor thapsigargin, did not block the action of raloxifene on neurons [49]. Thapsigargin causes a rapid increase in cytosolic Ca^2+^ concentrations via the specific inhibition of endoplasmic reticulum Ca^2+^-ATPases. In whole-cells, the amplitude of the high-voltage-activated Ca^2+^ current was significantly reduced by raloxifene (10 µM); however, raloxifene had no effect on NMDA-evoked Ca^2+^ current. Thus, raloxifene acutely reduces glutamate-induced intracellular Ca^2+^ increase, apparently via inhibition of high-voltage-activated calcium channels, but not by antagonism on estrogen receptors of neurons [49]. Thus, from the various studies discussed above, it appears that, depending on the context, raloxifene can regulate calcium currents potentially via estrogen receptors, CB-2 receptors, and high-voltage-activated calcium channels. What this may mean for cognition and neurorepair in whole animals needs to be investigated further.

To summarize from above, the SERM raloxifene can affect neuronal activities of various kinds associated with cognition and behavior. Underlying mechanisms include, but are not restricted to, neurotransmitter effects, regulation of gap junctions, and modulation of calcium homeostasis. 

### 3.2. Astrocytes

Astrocytes are specialized glial cells, outnumbering neurons over fivefold. While neurons can be considered to form the input–output signaling circuitry of the brain, astrocytes can be considered to form the supportive system embedding the neurons throughout the entire CNS. After brain injury, astrocytes acquire a reactive phenotype, including a series of morphological and molecular modifications, such as the expression of the cytoskeletal protein vimentin, which can be down-regulated by estradiol [50]. In general, astrogliosis (also known as astrocytosis or reactive astrocytosis) is part of the brain’s response to injury and disease. Astrogliosis can be presented as an enhanced increase in the number of astrocytes. Further, this astro-reactivity is characterized by morphological and functional changes. Thus, for one, astro-reactivity (under its various connotations) presents astrocytic responses to destruction of nearby neurons caused by CNS trauma, infection, ischemia, stroke, autoimmune responses, and neurodegenerative diseases [51,52]. Furthermore, two types of reactive astrocytes, termed A1s and A2s, can be discerned. On the one hand, neurotoxins released by A1 astrocytes can induce rapid cell death of neurons and oligodendrocytes, whereas A2 astrocytes, on the other hand, promote neuronal survival and tissue repair [53]. Astrocytic responses seen in various neurodegenerative diseases present a complex topic in need of more research to achieve better understanding of their classification, functions, and effects [54]. Interactions of astrocytes with neurons and microglia are presented in schematic form in Figure 2.

Neuroinflammation is a feature of many brain disorders, including injury and disease. The activation of glial cells (microglia as well as astrocytes), including their regulation of release of pro-inflammatory cytokines and chemokines, is a normal response oriented to protect neuron health and viability. Astrocytes can release proinflammatory molecules, including interleukin-6 (IL-6) and interferon-gamma-inducible protein-10 (IP-10) [55]. However, excessive and chronic activation of glia cells may lead to neurotoxicity and may be harmful for neural tissue [56]. The possible anti-inflammatory effects of several SERMs were assessed in primary cultures of astrocytes derived from newborn mice astrocytes. These cultures were exposed to lipopolysaccharide (LPS), which is a bacterial endotoxin known to cause neuroinflammation [55]. LPS increased IL-6 and IP-10 mRNA levels in these primary astrocytes. Moreover, protein expression of IL-6 and IP-10 in the culture medium of these primary astrocytes was increased by LPS application. These effects of LPS were attenuated by estradiol and by the four SERMs tested in this study: raloxifene, tamoxifen, ospemifene, and bazedoxifene [55,56]. From these data on astrocytes, it was suggested that estrogenic compounds may ameliorate neuroinflammation under neurodegenerative conditions in the brain by counteracting astrocytic production and release of proinflammatory molecules [55].

In animal studies, the effects of the estrogenic compounds raloxifene, tamoxifen, lasofoxifene (CP-336,156), bazedoxifene (TSE-424), and 17β-estradiol on the hippocampus of adult ovariectomized rats were assayed after administration of the excitotoxin kainic acid [35]. Administration of kainic acid induced the expression of vimentin in reactive astroglia in this paradigm. Disappointingly, in this study, however, SERMs did not affect this vimentin immunoreactivity in the hilus of the hippocampus [35]. Later, in another model for brain damage, vimentin did appear to be a valuable marker [50]. 

As mentioned, excessive and chronic activation of glia cells may lead to neurotoxicity and may be harmful for neural tissue [56]. Mentioned above, after brain injury, astrocytes acquire a reactive phenotype including a series of morphological and molecular modifications, such as the expression of the cytoskeletal protein vimentin, which can be down-regulated by estradiol [50]. It was assessed whether the SERMs raloxifene and tamoxifen could have effects similar to estradiol regarding down-regulation of reactive astrogliosis typically caused by brain injury. In the study of Barreto et al. [50], fifteen days after ovariectomy or sham surgery, animals received a stab wound to the brain and treatment with these estrogenic compounds. Raloxifene and tamoxifen reduced reactive astrogliosis in all experimental groups, indicating that they are potential candidates for the control of astrogliosis after brain injury [50].

As maintenance of neuronal homeostasis is an important function of astrocytes, to enhance astrocytic functions in this respect would present promising therapeutic strategies for improvement of brain function otherwise impaired by injury and disease. Encouragingly, it was found in cell culture that application of 1 µM raloxifene as a pretreatment to glucose-deprivation of astrocytic cells results in increases in cell viability and attenuation of nuclei fragmentation [57]. These favorable effects are associated with raloxifene reduction of oxidative stress and preservation of mitochondrial function [57]. These results also suggested direct effects of raloxifene on mitochondria [57]. In short, the data presented in Section 3.2, Astrocytes, suggest that, apart from maintaining metabolic homeostasis, estrogenic targeting of the production and release of pro-inflammatory molecules by astrocytes is a promising venue to counteract brain inflammation associated with neurodegeneration that is part and parcel of brain injury and disease. It appears important to conduct further research explicitly taking into consideration effects of astrocytic functioning in association with changes in cognition and behavior due to brain disease and brain injury.

### 3.3. Microglia

The neuroinflammatory features of microglial cells entail their abilities of transformation in response to a pathologic event. This includes their acquisition of an amoeboid morphology, enabling their reactive responses to harmful stimuli. These responses include migration, proliferation, and phagocytosis [58]. Because microglia cells display immediate activation by challenges to the brain’s homeostasis, they can be distinguished as “sensors of brain integrity” [59]. Neurological disorders typically present activation of glial cells, including their release of pro-inflammatory cytokines and chemokines. This is the normal CNS response to protect neural tissue and is mainly regulated by microglia and astroglia [27,28,29,30,31,32,60,61] (Figure 2). However, while acute glial activation is protective by design, failure of this protective acute activity may consequently lead to chronic glial activation, i.e., to enduring inflammation of neural tissue, and even cell death. Two general phenotypes of activated microglia are recognized: the proinflammatory M1 microglia, whose classical pro-neuroinflammatory responses include expression of major histocompatibility complex class II (MHC-II) and the release of the cytokines IL-6, IL-12, (TNFα, (**TNF**, cachexin, or cachectin; once **named** as tumor necrosis factor alpha or **TNFα**). A second class of activated microglia is designated as anti-inflammatory M2 microglia, which show neuroprotective aspects, characterized by release of the cytokines (IL-4, IL-13), brain-derived neurotrophic factor (BDNF), and insulin growth factor (IGF) [62]. However, it should be borne in mind that actual microglial cell activation presents a large variety in cell responses and cell types [63]. Based on this adaptive nature of microglia, in interaction with the local microenvironment, it has also been recognized that microglia not only present capabilities of neuroinflammatory responses, but that microglia also support many other important physiological processes related to normal, healthy functioning, such as brain wiring and maturation, including the pruning of excessive synapses and/or influencing neurogenesis [64,65,66]. 

In one study, it was found that SERMs, but not 17β-estradiol, induced significant, concentration-dependent anti-inflammatory responses in rat primary microglial cells, and also in mouse N9 microglial cells [67]. These responses were reflected by nitric oxide (NO) and IL-6 secretion, as well as total IL-6 mRNA expression. In N9 microglia, raloxifene and tamoxifen inhibited the potentiation of LPS response induced by trichostatin A, a histone deacetylase (HDAC) inhibitor. Interesting for clinical considerations, when treatment with the SERMs raloxifene, tamoxifen, or ICI 182.780 was applied simultaneously with LPS exposure (and even up to 6 h later) in acute models of mouse and rat microglial cells, these SERMs provided anti-inflammatory responses. The raloxifene-induced protection of N9 microglia was associated with a reduction of LPS-induced DNA binding activity of (Activator protein 1 (AP-1; transcription factor) [67]. Surprisingly, raloxifene or tamoxifen pretreatment starting 30 h before LPS exposure did not provide any protection of N9 microglia. Further studies then progressed to a better understanding of the underlying mechanism, as described below. 

The two SERMs, raloxifene and tamoxifen, were tested in vivo to see whether they could modulate the activation of microglia induced by peripheral administration of LPS [68]. The findings supported the potential therapeutic role of estrogenic compounds as protective anti-inflammatory agents for the CNS [68]. In this study, MHC-II immunoreactivity was used as a measure to assess microglial activation in the white matter of the cerebellum [68]. Estradiol, raloxifene, and tamoxifen decreased microglia activation induced by LPS in male and ovariectomized female rats. In particular, tamoxifen reduced microglia activation at all doses tested [(0.5–2 mg/kg body weight (b.w.)]. Similarly, raloxifene also reduced this microglial activation at this dose range, except for the higher dose (2 mg/kg b.w.). In addition, raloxifene, per se, had a moderate pro-inflammatory activity in the brain of control female rats and its anti-inflammatory activity was partially impaired in female animals after 1 month of deprivation of ovarian hormones [68]. Spots of estrogen receptor ERα immunoreactivity were detected in the soma and cell processes of microglia. Treatment with LPS, estradiol, or tamoxifen induced an increase of ERα immunoreactive spots in the perikaryon of microglia. The results indicate that some estrogenic compounds decrease brain inflammation by a mechanism that may involve estrogen receptors expressed by microglia [68]. 

As discussed above, it is well known that brain injury induces microglia activation, including secretion of pro-inflammatory molecules, and when this micro glia activation is chronic, it can potentiate damage. Thus, therapy that can limit microgliosis presents an important target for attenuation of progressing brain damage after brain injury [16]. This was studied for brain injury in young (as well as aged) animals, where raloxifene and tamoxifen were applied at time points considered relevant to clinic situations. These time points are considered to be within a handful of hours after brain trauma [16]. In the mentioned study, volume fraction of MHC-II(+) microglia was estimated according to the point-counting method of Weibel within a distance of 350 μm from the lateral border of the wound, and cellular morphology was measured by fractal analysis [16]. Two experimental groups were studied: (1) young rats, ovariectomized at 2 months of age; and (2) aged rats, ovariectomized at 18 months of age. Fifteen days after ovariectomy, a stab wound was given to the brain accompanied by treatment with estrogenic compounds. Raloxifene and tamoxifen reduced microglia activation in both young and aged animals, indicative of the neuroprotective capabilities of these SERMs in response to brain trauma [16]. In another study, functional rescue by raloxifene after TBI was associated with reductions in the activation of inflammatory M1 microglia and enhancing activation of anti-inflammatory M2 microglia [1], This was accompanied by reduced neuropathology of the optic system [1], as discussed in more detail in Section 5.3.

Although steroid hormones, including 17β-estradiol, can protect neuronal cells by attenuating excess activation of microglia, their use in the clinic is controversial because of contraindications e.g., increased risk for endometrial and breast cancer [69]. The effects of SERMs as agonists or antagonists of estrogen receptors are dependent on the target tissue. As designed, this selectivity of synthetic SERMs reduces their adverse side effects in peripheral tissue. In this way, associated with their anti-estrogen action in reproductive organs, in contrast to 17β-estradiol, SERMs present a very low risk for cancer. Regarding their desired curative effects, raloxifene, and tamoxifen attenuated LPS induced increases of proinflammatory cytokines and chemokine expression in rat primary microglia cultures [69]. Furthermore, microglial-conditioned media pretreated with raloxifene or tamoxifen significantly attenuated cellular injury in SH-SY5Y (neuronal) cells otherwise elicited by microglial-conditioned media treated with LPS alone. Rat primary microglia express the estrogen receptors ERα and ERβ primarily in the cell nucleus. The suppressive effects of raloxifene and tamoxifen on microglial activation was prevented by pretreatment with the pure estrogen receptor antagonist ICI 182,780. ICI 182,780 also prevented their protective action on SH-SY5Y cells [69]. A luciferase assay using a vector with three estrogen response elements (EREs) revealed that raloxifene and tamoxifen activated ERE-mediated transcription in rat primary microglia. These results suggested that the SERMs raloxifene and tamoxifen suppress microglial activation and subsequent neuronal cell death via an estrogen receptor-mediated transcription pathway [69]. In conclusion, the data presented above in this Section 3.3, Microglia show these SERMs’ ability to suppress neuroinflammation. This indicates that SERMs, such as raloxifene and tamoxifen, are promising candidates for treatments of disorders of the CNS. Figure 2 schematically presents interactions of microglia with neurons and astrocytes, including the microglial transition from resting stage to activated stage, in relation to their responses to brain disease and brain injury.

### 3.4. Oligodendrocytes (Myelination)

As said, SERMs appear to affect neurons, astrocytes, and microglial cells. Thus, it is also interesting to know whether and how SERMs may affect oligodendrocytes. For example, the effects of the antiestrogen keoxifene on the ontogenetic process of myelination were studied in male Zebra finches [33]. Keoxifene led to inhibition of myelination in the song motor center of the robust nucleus of the archistriatum (RA) and in the cerebellum [33]. Thus, most likely, raloxifene can also act on myelination. This may have implications for multiple sclerosis (MS). Associated with multifocal demyelination, this autoimmune disease of MS presents inflammatory cell infiltration of the CNS. Estrogen has beneficial effects regarding relapse-remittance of MS patients as demonstrated by clinical data and clinical indicators. To better understand the mechanism(s) of action of estrogen and estrogen receptor ERα underlying these beneficial anti-inflammatory effects, Hu and Qin [70] studied an experimental autoimmune encephalomyelitis (EAE) mouse model of MS. In their study, an ERα recombinant lentivirus was used to infect neurons with ERα. EAE was induced with the myelin oligodendrocyte glycoprotein (MOG) 35–55 peptide. The EAE mice were divided into five groups: an estrogen group (treatment with estradiol); an ERα agonist group (treatment with raloxifene); an ERα recombinant lentivirus group (ERα group, treatment with ERα recombinant lentivirus); an empty virus group; and a normal saline (NS) group. EAE was successfully reduced by stereotaxically injecting ERα recombinant lentivirus into the lateral ventricle of C57BL/6 mice. First of all, the results showed that the ERα recombinant lentivirus infection successfully enhanced ERα mRNA and ERα protein expression levels in the targeted neurons. This overexpression of ERα apparently inhibited inflammatory cell CNS infiltration, and decreased expression levels of Matrix metallopeptidase 9 (**MMP-9**), tumor necrosis factor-α (TNF-α), interferon-γ (IFN-γ), interleukin (IL)-17 and IL-23 expression levels, while expression of IL-4 was increased. In addition, overexpression of ERα reduced EAE incidence, and reduced nerve fiber demyelination, together with an increase in myelin basic protein (MBP) expression levels [70]. Moreover, the associated clinical symptoms were ameliorated. In conclusion, these data demonstrate that, similar to the effects of estradiol and raloxifene, overexpression of ERα, by using a recombinant lentivirus, ameliorates EAE in a mouse model. Thus, estrogen and raloxifene inhibit inflammatory responses, and raloxifene also alleviates damage to the myelin sheath. Collectively, ERα appears as a target for therapies of MS, other brain diseases, and injuries, and the known related functions and mechanisms.

### 3.5. Endothelial Cells

Endothelium is a single layer of squamous endothelial cells that line the interior surfaces of blood and lymph vessels. Thereby, the squamous endothelial cells form an interface between the lumen of blood and lymph vessels and the tissues they vascularize. In the brain the endothelium forms the blood–brain barrier (BBB), which serves to prevent damaging entities, such as toxins, viruses, and bacteria potentially present in blood circulation, to enter from the blood circulation into the brain tissue proper (i.e., the neurons with the neuropil). The endothelium has emerged as a key regulator of vascular homeostasis, as it does not only act as a barrier, but also functions as an active signal transducer [71]. 

Mitochondrial reactive oxygen species (ROS) and endothelial dysfunction are key contributors to cerebrovascular pathophysiology. As a mechanism to ameliorate such pathology, 17β-estradiol can enhance mitochondrial efficiency of energy production and suppress mitochondrial oxidative stress in cerebral blood vessels [71]. Cultured human brain microvascular endothelial cells (HBMECs) were used to determine whether estrogen acted specifically via endothelial estrogen receptors [71]. First, it was shown that increases in mitochondrial cytochrome c protein and mRNA expression were induced by treatment with 17β-Estradiol for a period of 24 h. Then applying silencing RNA for estrogen receptors showed that the estrogen receptor ERα was required for these effects, while ERβ appeared to be not involved. Measuring the activity of aconitase, which has an iron-sulfur center that can be inactivated by mitochondrial superoxide, was used to indicate mitochondrial ROS levels. The 17β-estradiol increased mitochondrial aconitase activity in HBMECs, indicating a reduction in ROS levels [71]. Furthermore, with the use of MitoSOX Red for direct measurement of mitochondrial superoxide, it was shown that mitochondrial superoxide production was significantly decreased by 17β-estradiol, but not by 17α-estradiol. This 17β-estradiol effect was blocked by the estrogen receptor antagonist, ICI 182,780. Selective estrogen receptor agonists also demonstrated that the decrease in mitochondrial superoxide was mediated by ERα, but not ERβ [71]. Raloxifene and 4-hydroxy-tamoxifen differentially affected mitochondrial superoxide production, with raloxifene acting as an estrogen receptor agonist and 4-hydroxy-tamoxifen acting as an estrogen receptor antagonist. Thus, estrogen receptors in endothelial cells also appear to present an estrogenic target that can serve to treat brain injury and brain disease with raloxifene.

### 3.6. Summary of Interactions of Raloxifene with Neural Cells

It appears that raloxifene can modulate cell specific functions of the various cell types in the brain. These cell types interact in intricate ways after brain injury and brain disease. Figure 2 presents various interactions between neurons, astrocytes, and microglia. This may explain why a compound, such as raloxifene, which apparently can modulate cell specific activities of all of these different cell types, may display quite effective overall ameliorating effects after brain injury and brain disease. For example, these cell activities have been found to include modulating neurotransmitter effects, cell death, plasticity, regulation of gap junctions, modulation of calcium homeostasis, myelination, oxidative stress, inflammatory responses, etc.

## 4. Intracellular Mechanisms (Nongenomic and Genomic) Modulated by Raloxifene

In the introduction, it was mentioned that SERMs can exert their actions via genomic functional pathways (by way of the cell nuclear estrogen receptors ERα and ERβ), including modulation of gene transcription. In addition, SERMs can also modulate intracellular mechanisms via various nongenomic pathways [15]. Moreover, in another relatively early study, it was suggested that both nongenomic and genomic pathways could be conducive for the possible protective effects of estrogen against neurodegeneration and cerebral ischemia. Genomic pathways, via the activation of cell nuclear steroid receptors, and nongenomic pathways, for example, via modulation of a plasma membrane steroid receptor, and/or a neurotransmitter, and/or by direct antioxidative action [72]. Moreover, the cell studies presented above implied that functional pathways affected by SERMs, such as raloxifene, can include modulation of various nongenomic functions, as well as via cell nuclear gene expression via estrogen receptor ERα and ERβ.

**Figure 3:**
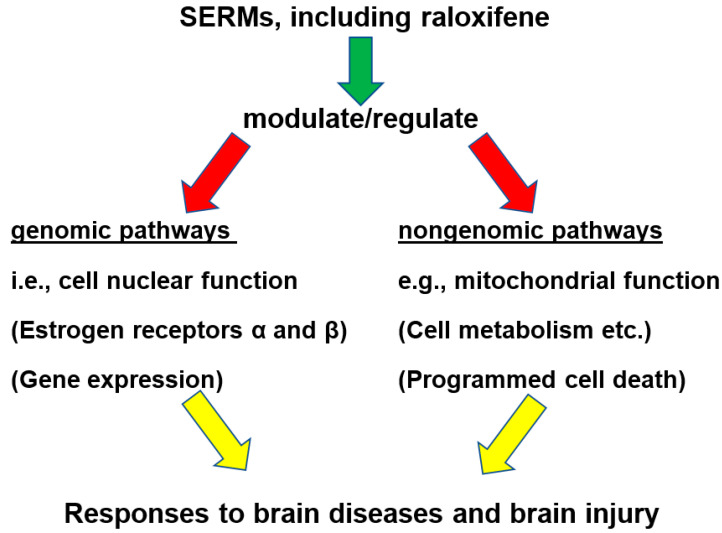
A generalized scheme of pathways, whereby SERMs, including raloxifene, may provide neuroprotection and curation in response to brain disease and brain injury. This is excerpted from the notion that SERMs, including raloxifene, appear to simultaneously affect genomic and nongenomic functions, i.e., cell nuclear function via the estrogenic receptors ERα and ERβ, modulating gene expression (genomic), while the various nongenomic functions include, but are not restricted to, interaction with the cell plasma membrane estrogen receptor (GPER), mitochondrial functions, cell metabolism, programmed cell death, as well as various others (e.g., [73,74,78,79]).

In this Section 4, it is explored, in more detail, for raloxifene, in association with its neuroprotective effects, whether the effects of this and other SERMs, indeed, include mitochondrial functions, in particular related to oxidative stress (i.e., nongenomic), as well as cell nuclear functions, i.e., modulation of cell nuclear gene expression (i.e., genomic). One study regarding activities of estradiol, as well as raloxifene, strongly suggested interactions between cell nuclear and mitochondrial functional pathways [73]. In this context, it was discussed that targeting estrogen receptors in endothelial cells may present a venue to treat brain injury and brain disease [73]. These can be cell nuclear estrogen receptors (ERα and ERβ), i.e., genomic as well as plasma membrane estrogen receptors (G protein-coupled estrogen receptor 1; GPER1, also named GPR30), i.e., nongenomic. This notion is schematized in Figure 3 toward the end of Section 4. Observations of gender-related differences in migraines also point to nongenomic and genomic functions of sex hormones [74]. 

### 4.1. Mitochondria (Including Oxidative Stress)

It has been shown that estradiol and raloxifene can affect the levels of nitric oxide (NO) and antioxidant enzymes in the brain cortex of ovariectomized female rats [72]. First of all, ovariectomy caused a decrease in total nitrite–nitrate levels. However, estrogen and raloxifene treatment induced higher NO levels than seen in the placebo group. Estradiol and raloxifene treatment had no statistically significant effect on superoxide dismutase (SOD) activity. Thus, activation of steroid receptors appeared to be a likely pathway for protection by raloxifene against depletion of NO, but in this study of Oge et al. [72] there was no effect on ROS generation.

Further investigations, regarding the effects of estradiol and raloxifene on antioxidant enzyme (SOD) and catalase (CAT)] activities as well as malondialdehyde (MDA) levels in brain and liver of ovariectomized female rats were done [75]. Ovariectomy lead to an increase in CAT activities in liver tissue samples and an increase in malondialdehyde (MDA) levels in brain of ovariectomized rat. Raloxifene treatment was able to reverse MDA levels in brain to normal levels. Based on these data, it can be concluded that raloxifene exerts anti-oxidative effects in brain [75]. Thus, raloxifene was suggested for treatment and/or prevention of diseases that can result from oxidative stress in postmenopausal women. Moreover, comparing the study of [75] with the study of [72], we can see that slightly differing paradigms can give differences in the results. However, taken together, it appeared, at that point in time that raloxifene moderately affected oxidative stress.

Konyalioglu et al. [76] further intensified the investigations on the effects of raloxifene on antioxidant enzymes such as SOD, CAT, and glutathione peroxidase *(GPx)*, on thioredoxin reductases *(TrxR),* and the levels of glutathione (GSH) and MDA in the heart, liver, and brain cortex of ovariectomized female rats. Significant increases in SOD, GPX, CAT activity, and MDA levels in brain, heart, and liver tissues could be induced by ovariectomy [76]. Such increases of SOD activity in the heart, GPX activity in the brain, and CAT activity in the liver could be counteracted by raloxifene treatment. The same raloxifene treatment of ovariectomized rats also significantly reduced MDA levels in the brain and heart (otherwise increased by ovariectomy), while raloxifene treatment was observed to significantly increase the levels of GSH in brain and heart tissues (otherwise decreased by ovariectomy) [76]. In conclusion, these results indicated that raloxifene may be effective against oxidative stress in brain and heart.

One later study strongly suggested interactions between cell nuclear and mitochondrial functional pathways regarding activities of estradiol as well as raloxifene [73]. In some detail, as discussed by Razmara et al. [73], it was known for cerebral blood vessels that 17β-estradiol profoundly affects mitochondrial energy production and suppresses mitochondrial oxidative stress. In cultured human brain microvascular endothelial cells (HBMECs), 17β-Estradiol treatment for 24 hours increased expression of mitochondrial cytochrome c protein and mRNA [73]. Reducing estrogen receptor expression by applying silencing RNA demonstrated the involvement of ERα, while ERβ did not appear to be involved. Aconitase is an enzyme with an iron-sulfur center that is inactivated by mitochondrial superoxide. Moreover, 17β-Estradiol increased mitochondrial aconitase activity in HBMECs, indicating a reduction in ROS [73]. By applying MitoSOX Red for direct measurement of mitochondrial superoxide, it was shown that 17β-estradiol, but not 17α-estradiol, significantly decreased mitochondrial superoxide production. This effect was blocked by the estrogen receptor antagonist, ICI 182,780. The SERMs, raloxifene and 4-hydroxy-tamoxifen, differentially affected mitochondrial superoxide production, i.e., raloxifene acts as an agonist decreasing mitochondrial superoxide production, while 4-hydroxy-tamoxifen acts as an estrogen receptor antagonist increasing mitochondrial superoxide. Thus, targeting estrogen receptors in endothelial cells may present part of the venue for treatment of brain injury and brain disease by raloxifene [73]. Using selective ER agonists also showed that the decrease in mitochondrial superoxide was mediated by ERα, and not by ERβ [73]. The authors presented the concluding remark: “Mitochondrial protective effects of estrogen in cerebral endothelium may contribute to sex differences in the occurrence of stroke and other age-related neurodegenerative diseases” [73]. Thus, from these studies it is clear that close interactions between cell nuclear and mitochondrial activities regarding of estradiol take place [73]. It was postulated previously that simultaneous modulation of cell nuclear and mitochondrial activity may present a characteristic of efficacious curative agents for neurodegeneration [28,29]. This review indicates that this appears to be also true for the SERMs, raloxifene, and 4-hydroxy-tamoxifen. 

Moreover, in another study, it was observed that estradiol, raloxifene, and tamoxifen administrations were beneficial on mitochondrial oxidative stress, inflammation, and poly (ADP-ribose) polymerase (PARP) levels in the serum and brain of ovariectomized rats by modulating antioxidant systems, DNA damage, and cytokine production [77]. Oxidative stress and mitochondrial dysfunction are major participants in various neurological disorders, where neuronal cells are severely affected by a decreased glucose supply to the brain [57]. Moreover, in astrocytic cell culture, glucose-deprivation resulted in nuclei fragmentation. It was found that 1 µM of raloxifene attenuated this nuclei fragmentation together with an increase in cell viability [57]. Furthermore, raloxifene may have direct effects on mitochondria, as suggested by its reduction of oxidative stress and preservation of mitochondrial function in glucose-deprived astrocytic cells [57].

Cell nuclear estrogen receptors have been the subject of estrogenic research for many years, together with estrogen receptor in the plasma membrane, as well as non-receptor effects of SERMs, thus, both genomic and nongenomic effects of SERMs are implicit in mainstream estrogenic research e.g., [73,74,78,79]. In the scheme of Figure 3, toward the end of Section 4, genomic and nongenomic effects of SERMs in relation to brain injury and brain disease are summarily outlined. While Section 4.1 focused on mitochondria and oxidative stress affected by SERMs, the next section, Section 4.2, pays attention to cell nuclear gene expression.

### 4.2. Cell Nuclear Gene Expression

Focusing more on cell nuclear gene expression modulated by raloxifene, several studies have shown that estrogens and SERMs, including raloxifene, can modulate gene expression of various proteins. For a review, see Carroll [80]. The following studies discussed here also show this to be true for the brain and brain cells. For example, in the hippocampus, estrogen regulates gene transcription underlying synaptic plasticity, neuronal development, neurophysiological functions, neuroprotection, memory consolidation, and behavior. This occurs, probably, via estrogen binding to ERα and ERβ [81]. In a study to characterize the estrogen receptor agonist/antagonist profile of raloxifene in the hippocampus and other brain regions of rats, its effect on the activity of choline acetyltransferase (ChAT) was examined in 6-month-old ovariectomized Sprague–Dawley rats [82]. ChAT activity decreased by approximately 20–50% in the hippocampus of ovariectomized rats compared to sham-operated control rats. Raloxifene and estradiol benzoate reversed the ovariectomy-induced decrease in ChAT activity in the hippocampus. This demonstrated the estrogen-like beneficial properties of raloxifene on hippocampal ChAT activity. 

Glutamate is the most important excitatory neurotransmitter in the CNS. Essential for brain function and brain damage, interactions between raloxifene and the glutamatergic system appear to involve modulation of expression of several proteins essential for various brain functions [48]. Raloxifene increased the expression of these proteins as well as neuronal survival [48]. In this study [48], glutamate dose-dependently regulates calcium oscillations, while raloxifene protected neurons from destruction by glutamate, and at the same time halted the decrease in expression of the memory-associated proteins.

Furthermore, comparable to the effects of estradiol on the expression of the α-amino-3-hydroxy-5-methyl-4-isoxazolepropionic acid AMPA (and NMDA types of glutamate receptors in the brain, regional agonist estrogenic activity of raloxifene and tamoxifen was observed [83]. Briefly, ovarian steroid withdrawal by ovariectomy decreased NMDA receptor specific binding in the hippocampus, compared to intact rat values. Estradiol treatment counteracted this NMDA decrease in the hippocampus. Estradiol treatment also decreased AMPA binding in frontal cortex, striatum, and nucleus accumbens. Raloxifene and tamoxifen showed similar estrogenic activity regarding modulation of NMDA and AMPA receptors in the brain [83].

In the hippocampus of ovariectomized rats, the effects of the excitotoxin kainic acid and the SERM raloxifene on the level of apurinic/apyrimidinic endonuclease/redox factor-1 (APE/Ref-1) mRNA were measured with real-time polymerase chain reaction [84]. APE1/Ref-1 is a multifunctional protein that can be secreted from cells. In addition to its anti-inflammatory activity and modulation of redox status, APE1/Ref-1 is part of the DNA base repair pathway, affects several types of transcriptional factors, and thereby is essential for genomic stability [85]. The results showed that the levels of APE/Ref-1 mRNA increased significantly in the hippocampus of rats that were challenged with kainic acid and treated with raloxifene, compared to kainic acid challenged rats that were not treated with kainic acid. It is known that 17β-estradiol deficiency stimulates mitochondrial functions for promoting apoptosis and increasing ROS production [86]. The treatments with 17β-estradiol, raloxifene, and tamoxifen enhanced IL-4 levels in plasma and decreased Western blot measurements for brain poly (ADP-ribose) polymerase (PARP) in ovariectomized rats, while proapoptotic procaspase 3 and 9 activities were increased by these treatments [86]. PARP is involved in DNA repair, genomic stability, and programmed cell death [87]. SERMs, such as raloxifene and bazedoxifene, evoked caspase-3-independent neuroprotection in neocortical neurons and increased protein levels of ERα (66 and 46 kDa isoforms) and peroxisome proliferator-activated receptor gamma (PPAR-γ). In addition, bazedoxifene enhanced expression of ERα-regulated aromatase (Cyp19a1) mRNA [88]. Aromatase is a cytochrome P450 monooxygenase that catalyzes the conversion of C19 androgens, androst-4-ene-3,17-dione (androstenedione), and testosterone to the C18 estrogens, estrone, and estradiol, respectively. Thus, raloxifene provides protection against the adverse effects of kainic acid, which includes modulation of expression of transcriptional factors, and proteins that are part of ROS generation, inflammatory pathways, and cell death [88]. 

Estrogen and SERMs can also modulate expression of serotonin receptors, transporters, and enzymes. It was shown that serotonin neurons of macaques express ERβ [89]. Estrogen treatment of female macaques via silastic implant altered gene expression for tryptophan hydroxylase (TPH), the serotonin reuptake transporter (SERT) and the 5HT1A autoreceptor [89]. Moreover, the SERMs, raloxifene and arzoxifene, acted in ways similar to natural estrogen on TPH and SERT mRNA expression in serotonin neurons [89]. In the monkey dorsal raphe, estrogen replacement and application of raloxifene also modulated TPH-1 mRNA; SERT mRNA and monoamine oxidase (MAO)-A mRNA. Thus, estrogen and raloxifene appear to increase serotonin production and transport to various extents. This, in combination with changes in expression of degradative enzymes, presents a complex of gene transcription, post-transcriptional processing, and substrate feedback mechanisms [90]. Sumner et al. [91] provided evidence that estradiol induction of the 5-HT(2A)R and the SERT in the brain is mediated by nuclear estrogen receptors. The SERM raloxifene completely blocked estradiol stimulation of 5-HT(2A)R and SERT expression in the brains of acutely ovariectomized rats [91]. In particular, in the mid-frontal cortex, raloxifene alone increased the density of SERT sites, while in the posterior olfactory tubercle it decreased the density of 5-HT(2A)R. 

Regarding the neurotransmitter dopamine, in ovariectomized rats 17β-estradiol and raloxifene modulated D2 and D3 specific binding in the basal ganglia [92]. This obviously may have implications for various dopamine related disorders, ranging from Parkinson’s disease to schizophrenia. Another neurotransmitter closely associated with basal ganglia disorders is enkephalin. The following study investigated the effect of hormonal withdrawal and replacement therapy on preproenkephalin (PPE) expression in the striatum and nucleus accumbens [93]. In this study, ovariectomized Sprague–Dawley rats were treated for 2 weeks with estradiol, specific ligands for ERα and ERβ (PPT and DPN respectively), and with the SERMs, raloxifene and tamoxifen. In the nucleus accumbens and striatum, PPE mRNA levels were decreased by ovariectomy, an effect that was counteracted by estradiol. PPT, DPN, tamoxifen, and raloxifene reproduced the estradiol effect in the accumbens [93]. The same effect was obtained with SERMs and the agonists for ERα and ERβ [93]. 

Effects of raloxifene were studied in the Mouse Motor Neuron-Like Hybrid Cell Line (**NSC**-**34**) model of amyotrophic lateral sclerosis (ALS) that stably expresses the 25-kDa C-terminal fragment of transactive response DNA binding protein **43** kDa (**TDP**-**43**) (i.e., TDP-25 cells). These TDP-25 cells express G protein-coupled receptor 30 (GPR30; also named GPER) as well as ERα and ERβ [94]. Located in the plasma membrane, GPR30 is a seven-transmembrane-domain receptor that mediates non-genomic estrogen related signaling [95]. Being a receptor, responding to estrogen and SERMs, GPR30 affects functional pathways serving to modulate cell growth, migration and programmed cell death in a variety of tissues. In the study of Zhou et al. [94] the expression of ERα and GPR30 were both increased by 17β-estradiol as well as raloxifene. Furthermore, 17β-estradiol as well as raloxifene enhanced TDP-25 cell viability. All of these effects of 17β-estradiol and raloxifene were completely abolished by the ERα/β antagonist (ICI 182,780) as well as the GPR30 antagonist (G15) [94]. P62, caspase-9, and Bax levels were significantly decreased in TDP-25 cells treated with 17β-estradiol or raloxifene, and levels of the lipid modified form of microtubule-associated **protein** 1A/1B-light chain 3 (LC3-II) were elevated in 17β-estradiol-treated cells, but reduced in raloxifene-treated cells. All of these changes were abolished by treatment with ICI 182,780 or G15 [94]. These data suggest that both raloxifene and 17β-estradiol enhance autophagy and suppress apoptosis to limit motor neuron death by binding to ERα/β and/or GPR30 in TDP-25 cells. The results in this ALS model indicate that the raloxifene provides its neuroprotective effects via genomic pathways as well as nongenomic pathways [94].

It is known that raloxifene not only binds to estrogen receptors, but also to CB-2 cannabinoid receptors, for examples, see [1,96]. Like the well-established CB-2 inverse agonists (AM630, JTE907), the CB-2 inverse agonist raloxifene acutely increased cortical c-Jun NH_2_-terminal kinase (JNK) levels in mice [96]. In more detail, AM630 acutely increased JNK in cortex (+61–148%); JTE907 and raloxifene also acutely increased cortical JNK (+31–57%). Repeated (i.e., chronic) treatments with raloxifene, such as with AM630 and JTE907, also brought a reversal to the acute effects even resulting in decreases in cortical JNK (AM630: −36%; JTE907: −25%; raloxifene: −11%). Thus, acute and chronic treatments with CB-2 inverse agonists (such as raloxifene) regulate cell death markers in opposite directions in the brain [96]. 

Thus, both sections together: 4.1 mitochondria (including oxidative stress) and 4.2 cell nuclear gene expression, show that raloxifene and other SERMs affect both mitochondrial activity and oxidative stress as well as gene expression, i.e., nongenomic and genomic functional pathways/mechanism. Figure 3 briefly summarizes that SERMs, including raloxifene, display these properties of modulation of both genomic and nongenomic functional pathways. This may be a general property of agents efficacious in treatment of brain disease and brain injury: namely, simultaneous effects on genomic and nongenomic functions, including cell nuclear gene expression and mitochondrial function. Effects on cell nuclear gene expression and mitochondrial function were also observed for other agents efficacious in treatments for neurodegeneration in animal models and cell culture, as reviewed, for example, for TSPO ligands and fucoidan [28,29]. These two sections presented here show that it is very informative to study simultaneous activities of mitochondria and cell nuclei. In particular, from our point of view, such research will lead toward novel treatments of neurodegeneration, as related to brain disease and brain injury. On a more general base, the various genomic and non-genomic functional pathways, and mechanisms under estrogen control, appear to make estrogens and SERMs potential broad-spectrum agents for treatment of brain injury and brain disease [6].

## 5. Brain Diseases, Brain Injuries, and Underlying Functions in Response to Raloxifene

### 5.1. Alzheimer’s disease, Dementia

**Figure 4:**
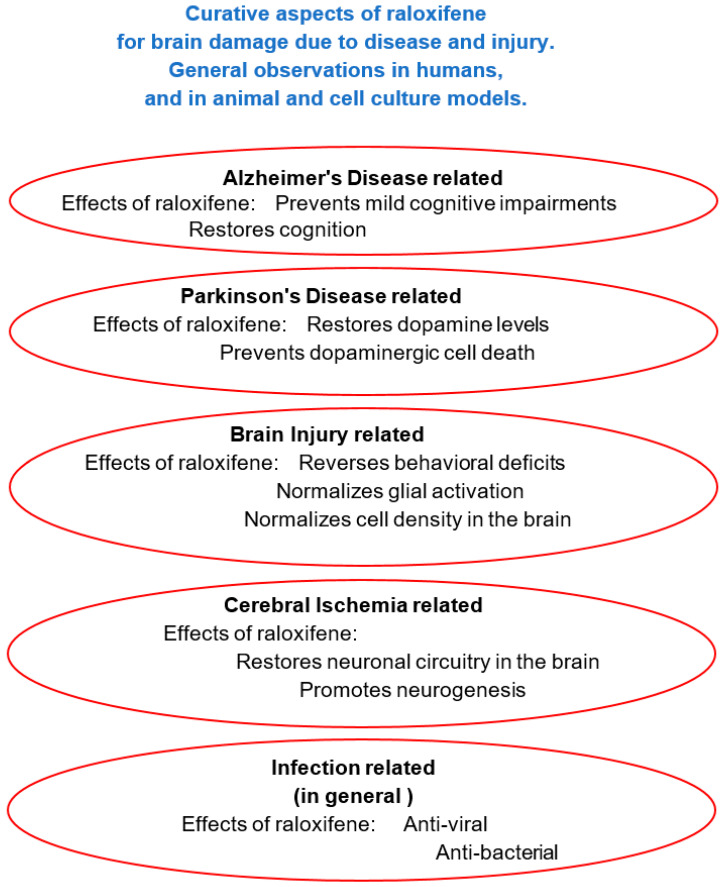
Beneficial effects of raloxifene regarding brain disease and brain injury. Note: each ellipse presents the typical experimental approach applied so far for that model. One can assume that, in principle, most effects seen with raloxifene can occur in each model.

Alzheimer’s disease is the most common cause of dementia, accounting for 60–80% of the cases, and is in dire need of therapy. Dementia is defined by memory loss and other cognitive impairments serious enough to interfere with daily life [97]. Alzheimer’s disease, discussed in this section (Section 5.1. Alzheimer’s disease, Dementia) includes cell and tissue loss throughout the brain. This neurodegeneration presents itself as shrinkage of the brain as a whole. In other brain disorders, such as Parkinson’s disease, ALS, MS, Stroke, and TBI, which also have been assayed for treatment with raloxifene and other SERMs (as discussed in Section 5.2, Section 5.3 and Section 5.4) the shrinkage, i.e., neurodegeneration, typically is restricted to localized brain areas and specific cell types. In Figure 4, the relevance of raloxifene for these brain diseases and brain injury is outlined schematically.

In 2002, 42 studies were reviewed to address the question whether estrogen replacement therapy (ERT) would be beneficial for memory and cognition performance in nondemented postmenopausal women [98]. An appreciable number of nonsignificant findings was acknowledged, importantly however, the number of significant findings favoring ERT users considerably outnumbered the rare findings of better performance in controls [98]. Experimental, short-term studies demonstrated a consistent beneficial effect on verbal memory of estrogen replacement therapy. Moreover, long-term observational studies suggested a long-lasting beneficial effect of continued estrogen replacement therapy on cognitive functioning. However, these long-term studies have to be interpreted with caution as they lacked random assignment [98]. Studies on the effects of ERT showed an association with a decreased risk for development of dementia, but no significant effects of ERT were seen with patients already diagnosed with Alzheimer’s disease [98].

A later study, a randomized, placebo-controlled trial, examined whether long-term application of raloxifene affected the risk for Alzheimer’s disease in postmenopausal women with osteoporosis [39]. The women who were the subjects of this part of the study received raloxifene (60 or 120 mg/day) and the effects of these treatments on development of mild cognitive impairment and dementia were evaluated [39]. These women received baseline and annual clinical and cognitive evaluations [39]. In particular, as described in this study by Yaffe et al. [39], after 3 years, among the 5386 women enrolled at participating sites, those who had clinical symptoms of dementia or scored in the lowest 10th percentile on cognitive screening were evaluated by a blinded dementia specialist and had brain scans and laboratory tests to evaluate dementia etiology. Dementia was diagnosed by a blinded adjudication committee. Of the 5386 women of the trial, 5153 (95.7%) were classified as cognitively normal, 181 (3.4%) had mild cognitive impairment, and 52 (1.0%) had dementia, 36 with Alzheimer’s disease. Compared to those taking placebo, women receiving 120 mg/day of raloxifene had a 33% lower risk of mild cognitive impairment (relative risk, 0.67; 95% confidence interval [CI], 0.46–0.98), and also lower risk of Alzheimer’s disease, the latter which, however, was non-significant (relative risk = 0.52, 95% CI = 0.22–1.21) [39]. Looking at these numbers, as reduction of risk for Alzheimer’s disease presents a larger number than the reduction of risk for mild cognitive impairment (48% vs. 33%), it appears that the effect of raloxifene on progressing to diagnosis of Alzheimer’s disease shows a relatively large variability. This is in accord with the earlier studies reviewed by Zec and Trivedi [98]. Moreover, 60 mg/day of raloxifene had no significant effects. Thus, raloxifene at a dose of 120 mg/day resulted in reduced risk of cognitive impairment in postmenopausal women [39]. Regarding later verbal memory studies, similar findings were reported. Raloxifene improved verbal memory of late postmenopausal women in a randomized, double-blind placebo-controlled trial [41]. Briefly, the women receiving raloxifene performed better than the women receiving placebo in the “words A + B test” (*p* = 0.025) and in the “words A test” (*p* = 0.023). These findings were solidified by a later review of the various trial studies done until 2013 [5]. Importantly, these finding are not without implications. Notably, patients with mild cognitive impairment convert from this diagnosis to a diagnosis of Alzheimer’s disease at an average rate of 15% per year [99]. With simple arithmetic, this implies that, from any time point of choice, within seven years, all patients with mild cognitive impairment will have progressed to full blown Alzheimer’s disease. Because it was shown for a considerable number of postmenopausal women that raloxifene may present protective effects regarding progress toward diagnosis of Alzheimer’s disease, as discussed, foreknowledge, which patients would show the positive outcome, would help to apply raloxifene prophylactically [100]. This is the more important, as raloxifene did not reliably treat Alzheimer’s disease itself, as found with subjects treated with raloxifene for osteoporosis. Moreover, a pilot study directed at Alzheimer’s disease per se in postmenopausal women did not show curative effects [101]. Applying a successful agent prophylactically to arrest the development progressing toward Alzheimer’s disease may also reduce the burden and distress of Alzheimer patient caretakers. The idea of applying raloxifene prophylactically is reminiscent of the prophylactic treatment with raloxifene to prevent the invasive type of breast cancer in women at high risk for invasive breast cancer [102]. It is also essential to make sure that effects of treatment will be ameliorating and not detrimental. Interestingly, it was also shown with invasive breast cancer that raloxifene slightly enhanced survival rate, i.e., was not detrimental in this respect [102]. 

For this purpose of prophylactic treatment to prevent occurrence of Alzheimer’s disease, of course it is important to be able to tell which patients, or even healthy persons, are at risk for Alzheimer’s disease. Interestingly, a study on glycosylated hemoglobin (HbA1C), a marker of glucose control, supported the hypothesis that glucose dysregulation is a predictor for cognitive impairment [40]. For this study, 1983 postmenopausal women (mean age, 67.2 years) with osteoporosis were assayed over a 4 year period. Further studies like these may indicate ways to determine for which persons raloxifene may present an efficacious treatment. First of all, of course, persons with mild cognitive impairment should get extra attention. Indeed, it is then important to know what are the defining characteristic of persons with mild cognitive impairment that may predict the further development toward Alzheimer’s disease. 

Moreover, molecular biological mechanisms whereby raloxifene might counteract development toward Alzheimer’s disease were explored. For example, the mechanisms were studied whereby desmosterol [3-β-hydroxysterol delta-24-reductase (DHCR24)], also named SELective Alzheimer’s Disease INdicator-1 (seladin-1), may be involved in the potential role of SERMs in their effects in Alzheimer’s disease [103]. Seladin-1/DHCR24 shows anti-apoptotic activity and catalyzes the synthesis of cholesterol from desmosterol. In particular, in neuroblastoma cells, the amount of membrane cholesterol was increased by Seladin-1 overexpression. In addition, Seladin-1 overexpression induced resistance against β-amyloid aggregates. In agreement, a specific DHCR24 inhibitor increased cell vulnerability [103]. Interestingly, 17β-estradiol, raloxifene, and tamoxifen significantly increased the expression of seladin-1 and the amount of cell cholesterol. This probably associates with their effects in human fetal neuroepithelial cells (FNC) where they protect against oxidative stress and β-amyloid toxicity [103]. Corroborating these findings, upon seladin-1 silencing in FNC cells, the protective effects of SERMs are abolished [104]. Thus, seladin-1 may be a mediator for the neuroprotective properties of SERMs and cholesterol’s function in maintaining brain homeostasis.

Interestingly, raloxifene also appears to be able to inhibit Aβ42 aggregation directly and to destabilize preformed Aβ42 fibrils by directly interacting with the N-terminus and middle domains of Aβ42 peptides [105]. Thus, raloxifene can directly reduce toxicity of Aβ42 in HT22 neuronal cells. Furthermore, in this way, raloxifene can also prevent expressions of tumor necrosis factor-α and transforming growth factor-β that are normally induced by Aβ42 peptides [105]. Thereby, also, microglia-mediated, indirect toxicity of Aβ42 to HT22 neuronal cells can be alleviated. This says that, apart from effects via receptors (genomic and non-genomic) and modulation/regulation of various molecular biological mechanisms (e.g., related to metabolism, oxidative stress, and others) raloxifene can present itself as a direct, substrate affecting agent. 

As mentioned, raloxifene may delay the onset of mild cognitive impairment in elderly women, and, as a consequence, may prevent or delay the development toward and the onset of Alzheimer’s disease. Thus, it was considered that functional magnetic resonance imaging (fMRI) may reveal effects of raloxifene on brain activation patterns during a memory task in postmenopausal women [106]. In this study, treatment with raloxifene resulted in reduced activation in the left parahippocampal and lingual gyri, and increased activation in the right superior frontal gyrus in this paradigm [106]. 

As raloxifene presented the hopeful finding that it may delay the onset of mild cognitive impairment in elderly women, it was also realized that is was mandatory to investigate potential beneficial effects of raloxifene treatment on mental performance in males [107]. For this purpose, functional magnetic resonance imaging (fMRI) was applied to measure brain activity in elderly males. This showed that brain activation associated with encoding of new information (faces) into memory was enhanced by raloxifene treatment. In more detail, raloxifene treatment enhanced brain activation in the left posterior parahippocampal area (Z = 3.9) and right inferior prefrontal cortex (Z = 3.5) [107]. Furthermore, while the placebo group showed a small but significant decrease in accuracy scores (*p* = 0.02), recognition accuracy scores remained stable in the raloxifene group. In conclusion, these findings of raloxifene’s positive effects on memory in men and women associated with raloxifene’s effects on brain activity [106,107]. It was argued that the validity of these predictions could be tested in large-scale clinical trials [107].

With recent advancements in imaging techniques, pharmacology, and molecular biology, the potential exploitation of these and other signaling pathways for clinical use is becoming possible. This would allow to further test the efficacy of raloxifene and other agents to slow or stop the development toward Alzheimer’s disease, and other neurodegenerative diseases [28,29,97]. 

To briefly mention something relatively new, it is becoming appreciated that infection (bacterial, viral, yeast) may play a role in the development of Alzheimer’s disease, and it has been proposed that TSPO ligands and fucoidan may counteract such infections [28,29,34,108,109]. Very interestingly, also this year, the SERMs raloxifene hydrochloride and quinestrol were among 15 compounds that were found to effectively inhibit Zika virus (ZIKV), Dengue virus (DENV), and West Nile Virus (WNV) (Kunjin strain) infection at low micromolar concentrations with minimal cytotoxicity in Huh-7.5 hepatoma cells and HTR-8 placental trophoblast cells [110]. Furthermore, these 15 compounds appear to inhibit viral RNA replication in a manner that is independent of their known effects on estrogen receptor signaling (ERα and ERβ) [110]. This may indicate that their effects are via one or more SERM associated nongenomic pathways. Taken together, raloxifene hydrochloride, quinestrol, and structurally related analogues warrant further investigation as potential therapeutics for treatment of virus infections, including related to brain diseases, such as the slow development toward Alzheimer’s disease. Again, it has to be realized and appreciated that SERMs, including raloxifene, rather prevent development of Alzheimer’s disease when applied relatively early during the development of mild cognitive impairments, but do not provide a cure for diagnosed Alzheimer’s disease once it has developed from mild cognitive impairment. Thus, speculating, raloxifene may stop an infection leading to Alzheimer’s disease in an early stage, but cannot overcome a full-blown infection and its aftermath damage. The study of Eyre et al. [110] also suggests that raloxifene does not combat all types of viruses. This may explain why raloxifene effects on development of Alzheimer’s disease may show the large variability that is seen [41,98,101]. In addition, to emphasize apparent anti-viral, as well as anti-bacterial properties of raloxifene, it has been found that it can also be used in treatment against malaria [111].

Possibly, a viral, bacterial, or yeast load in the brain may predictive for development of Alzheimer’s disease. If that is indeed the case, then by having a compound ready like raloxifene, fucoidan, or TSPO ligand, which separately or together may act against such an infection [28,29], hopefully Alzheimer’s disease can be stopped before it develops (just like invasive breast cancer can be prevented by raloxifene).

To recapitulate here, a major finding appears to be that raloxifene can counteract mild cognitive impairment, as pointed out in Figure 4. Disappointingly, clinical trials of postmenopausal women treated with raloxifene for osteoporosis, did not shown an effect on concomitant Alzheimer’s disease presenting itself in a number of these patients. Moreover, a pilot study directed at Alzheimer’s disease, per se, did not show curative effects [101]. Nonetheless, the findings regarding mild cognitive impairment are not without relevance, not only already in and by itself, but also because, in a relevant number of cases, diagnosis of mild cognitive impairment precedes diagnosis of Alzheimer’s disease. Thus, if (and when) it can be predicted which patients with mild cognitive impairment disease will, at a later stage, be diagnosed with Alzheimer’s disease, it will be prudent to prophylactically treat these patients. This review suggests raloxifene for such a treatment, but of course, also, other agents with similar properties may be just as effective, or even better. This would, hopefully, also reduce caretaker burden and caretaker distress. Finally, the various studies in this section also are in agreement with the general notion that raloxifene affects cell nuclear function of gene expression as well as various other mitochondrial and non-mitochondrial functions from metabolism to programmed cell death and even modulation of cholesterol production and degradation of Aβ1-42, i.e., genomic and nongenomic pathways and mechanisms (see Figure 3).

### 5.2. Parkinson’s Disease

In contrast to Alzheimer’s disease and raloxifene, which entail various human studies, so far for Parkinson’s disease studies only animal models (rodents) have been applied to determine effects of raloxifene and other SERMs. Perhaps this is so because rodent models for Parkinson’s disease are relatively robust. Regarding humans, epidemiology documented that the prevalence of Parkinson’s disease is higher in males than in females. This suggested that estrogens may be involved, maybe even beneficial. This then was studied in animal models for Parkinson’s disease, for example, by dopamine depletion in striatum in C57Bl/6 mice induced by 1-methyl-4-phenyl-1,2,3,6 tetrahydropyridine (MPTP). In the study in question here, this was done by giving four systemic (intraperitoneal; i.p.) injections of MPTP, 15 mg/kg i.p. at 2 h intervals [104]. Interestingly, 17β-estradiol, progesterone, and raloxifene, can prevent this MPTP induced dopamine depletion, whereas 17α-estradiol had no effect. Mechanisms how this may occur were assayed [104]. Integrity of dopamine neurons was probed by dopamine transporter (DAT) labeling {labeled by [(125)I]RTI-121 specific binding}. For treatments, 17α-estradiol, 17β-estradiol, or progesterone, were injected daily for 5 days prior to the MPTP injections and then these estrogenic injections were continued for 5 days after the MPTP injections. In the striatum, the MPTP injections resulted in a decrease of DAT-specific binding (50% of control). In the substantia nigra, these MPTP injections reduced DAT mRNA levels (20% of control). The 17β-estradiol (2 µg/day), progesterone (2 µg/day), and raloxifene (5 mg/kg/day) prevented these MPTP induced decreases in the striatum, but 17α-estradiol (2 µg/day) or the lower dose of raloxifene (1 mg/kg/day) did not [104]. In the substantia nigra, none of these mentioned treatments completely reversed the decrease of DAT mRNA levels. Nonetheless, 17β-estradiol, as well as progesterone and raloxifene treatment, provided protection against this MPTP neurotoxicity [104]. Thus, the early dopamine nerve cell damage modeled in this paradigm was responsive to hormone treatment. Many other studies also showed that the neuroprotective mechanisms of estrogens include antioxidant effects and upregulation of Bcl-2, brain-derived neurotrophic factor (BDNF), and glial cell-derived neurotrophic factor (GDNF) [112]. In addition to transcription regulation by cell nuclear estrogen receptors, upregulation of Bcl-2 or GDNF is mediated by nonnuclear estrogen receptor [112]. This also indicates that genomic–nongenomic interactions take place.

In another experimental study, 17β-estradiol also inhibited MPTP-induced dopamine depletion by applying a dosing regimen of 17β-estradiol that mimics physiological levels of this steroid [113]. However, high doses of 17β-estradiol failed to inhibit toxicity, as did 17α-estradiol. Disappointingly, however, in this paradigm raloxifene was ineffective against MPTP toxicity [113]. Thus, it was pointed out that exploration of new compounds with different pharmacological and/or physiochemical properties maybe warranted [113]. 

Fortunately, however, studies by others showed prevention of MPTP-induced striatal dopamine depletion in mice by 17β-estradiol, progesterone, and by raloxifene (for example, [114]). To get better insights into the neuroprotective effects of raloxifene in MPTP-treated male mice, its mediation via G protein-coupled estrogen receptor 1 (GPER1) was studied. GPER1, in contrast to the cell nuclear ERα and ERβ estrogen receptors, is located at the plasma membrane. To study the influence of GPER1, its antagonist G15 was used [115]. Striatal concentrations of dopamine, 3,4-dihydroxyphenylacetic acid, homovanillic acid to dopamine ratio as well as dopamine transporter and vesicular monoamine transporter 2, after MPTP, showed that raloxifene neuroprotection of dopaminergic neurons was blocked by G15. Mechanisms of protection by raloxifene were found to include striatal Akt signaling. (Activation of Akt signaling promotes cell survival, cell growth, cell proliferation, cell migration, and angiogenesis.) Furthermore, the protection by raloxifene was accompanied by increased Bcl-2 and brain-derived neurotrophic factor (BDNF) levels; these effects were also abolished by co-administration with G15. This study indicates that to achieve protection of dopaminergic neurons in MPTP treated mice, raloxifene acts by way of GPER1 to mediate Akt activation, and to increase Bcl-2, and BDNF levels, thereby maintaining plasma androgen levels.

Finally, in another model for Parkinson’s disease, nigral dopaminergic cell death in Wistar rats was induced by striatal injections of 6-hydroxydopamine (6-OHDA). In this model, 17β-estradiol and the SERMs, raloxifene and tamoxifen, as well as a selective estrogen receptor REα agonist, propyl-pyrazole-triol (PPT) and a selective estrogen receptor REβ agonist, diarylpropionitrile (DPN), were assessed for their effects on behavioral and biochemical alterations. Not only did striatal injection of 6-OHDA induce significant behavioral alterations reminiscent of Parkinson’s disease, these effects were accompanied by significant decreases in striatal dopamine, homovanillic acid (HVA), and 3,4-dihydroxyphenyl acetic (DOPAC) concentrations [116]. Furthermore, 6-OHDA-induced nigral acid dopaminergic cell death that was accompanied by oxidative stress, presented as a significant decrease in striatal glutathione peroxidase (GPx) activity, as well as apoptosis in the substantia nigra, the latter as indicated by a significant increase in nigral caspase-3 activity [116]. Treatment with 17β-estradiol, raloxifene, and PPT, but not with tamoxifen or DPN, provided significant ameliorations of the behavioral and biochemical effects induced by 6-OHDA [116]. Thus, it appears that specific SERMs, including raloxifene, may be very effective for treatment in the 6-OHDA induced rat model for Parkinson’s disease, reminiscent of the MPTP mouse model for Parkinson’s disease.

In addition to the effects on depletion of dopamine by the MPTP and 6-OHDA models for Parkinson’s disease, and the beneficial effects of SERMs, including raloxifene, inhibition of dopamine activity by cocaine was also explored. Estrogenic effects included restoration of dopamine activity otherwise inhibited by cocaine. We focus here, quickly, on the effects of raloxifene. Pretreatment with this estrogen receptor antagonist raloxifene or the selective ERα antagonist Y134 largely counteracted cocaine-inhibition of dopamine neuron firing [117].

In short, raloxifene appears to have beneficial effects in respect to dopamine depletion and reduction of dopamine activity in the MPTP and 6-OHDA models for Parkinson’s disease, as pointed out in Figure 4. 

### 5.3. Traumatic Brain Injury (TBI)

In addition to the search for beneficial effects of raloxifene treatment on Alzheimer’s disease and Parkinson’s disease, over the years, raloxifene also received attention in relation to brain injury. In this search for effective treatments in experimental models for TBI, hormonal differences between males and females also appeared to be a crucial component. In one study, bilateral cortical contusion injury (bCCI) or sham procedure was applied to male rats to examine functional recovery effected by raloxifene treatment [118]. Injections of raloxifene (3.0 mg/kg, i.p.) or vehicle (1.0 mL/kg, i.p.) were given 15 min, 24, 48, 72, and 96 h after bCCI or sham procedure. Sensorimotor tests (bilateral tactile removal and locomotor placing tests) and cognitive tests (reference and working memory in the Morris water maze) were applied to see the effects of raloxifene-treatment in comparison to vehicle-treatment. Raloxifene significantly reduced the initial magnitudes of the deficits and accelerated recovery [118]. Interestingly, even though within this timeframe of 96 h, raloxifene treatment did not present a significant reduction in the lesion size of the TBI, nevertheless, raloxifene treatment apparently improved functional outcome following bCCI.

In another paradigm, fifteen days after ovariectomy or sham surgery, rats received a stab wound injury to the brain together with treatments applying estradiol, raloxifene, or tamoxifen [50]. Reactive astrocytes after such stab wounds were more numerous in aged rats than in young rats. Nonetheless, in both age groups, the estrogenic compounds reduced reactive astrogliosis [50]. Raloxifene and tamoxifen also reduced microglia activation after stab wound brain injury [16]. These data suggest that early regulation of microglia activation, in addition to regulation of astrogliosis, provide a functional pathways by which SERMs may exert neuroprotective effects in the setting of a brain trauma [16,50]. These studies also indicate simultaneous effects on astrocytes and microglia regarding neurodegeneration and neuroprotection as outlined in Figure 2.

Various visual deficits after TBI are common and thus can be used for assays. For example high pressure closed-head air blast yields deficits in visual acuity and contrast sensitivity, reductions in the A-wave and B-wave of the scotopic electroretinogram (ERG), light aversion, and increased pupil constriction to light [1]. Raloxifene (at 5–10 mg/kg) delivered daily for two weeks after closed-head air blast TBI mitigates or eliminates mentioned visual abnormalities (the higher dose typically being more effective). As mentioned, this functional rescue by raloxifene was associated with reductions in the activation of inflammatory M1 microglia and enhancing activation of anti-inflammatory M2 microglia, as well as with reduced damage and neuropathology of the retina, optic nerve, and oculomotor nucleus [1]. 

Interestingly, for these beneficial raloxifene effects, interactions with the cannabinoid type-2 receptor (CB-2) were essential [1]. In other experiments, treatment with the CB-2 inverse agonist SMM-189 for 2 weeks after closed-head blast TBI greatly attenuated the visual deficits and retinal pathology otherwise produced in mice, including reduction of the deleterious role of microglia in the injury process after this trauma [23,24,25,26,119]. Thus, in general, the CB-2 receptor provides a robust pathway for healing of brain injury. A practical advantage of raloxifene over SMM-189 in this respect is that SMM-189 as yet lacks FDA approval, while raloxifene has been approved and applied, as well as studied in human disorders for more than two decades (as this review bears witness, not only clinical trials, but also including numerous animal experiments during this time period). Moreover, important for potential clinical application, including clinical trial, raloxifene treatment was shown to be still effective even when delayed until 48 h after TBI [1]. This would mean that definitely sufficient time is available to bring victims/patients to the clinic, for diagnosis, and for clinical trial to obtain informed consent. And of course, hopefully, eventually also for actual treatment with raloxifene of TBI patients. Summarizing, the various studies show that raloxifene is beneficial for treatment of brain injury. Moreover, this is also true for other brain disorders presented in this review. Thus, these results, plus raloxifene’s regular use in humans for other health problems, provide a safe and sound basis for clinical trial, maybe even phase-2 efficacy testing targeting treatment of TBI by raloxifene in humans [1]. In addition, the body of research on TBI treated with raloxifene also shows that raloxifene ameliorates the pathologies of neurons, including axons, and of astrocytes and microglia, which all are typical for neurodegeneration (Figure 2), presenting raloxifene as a broadband curative drug for TBI. The latter may mean that raloxifene should also be effective for treatment of other brain disorders. 

### 5.4. Cerebral Ischemia

To recapitulate, important for their effectiveness of treatments of brain disease and brain injury, SERMs promote neurorepair, as well as neuroprotection together with anti-neuroinflammatory effects, and ameliorate behavioral impairments, as discussed above, for various brain injuries and diseases, including the effects on the various types of brain cells. Now, regarding cerebral ischemia, in a detailed study it was investigated whether acute regulation of neurogenesis and spine remodeling could be part of amelioration of damage due to cerebral ischemia [46]. Toward this end, ovariectomized adult female rats received injections of raloxifene, or implants of pellets containing tamoxifen or 17β-estradiol (estrogen). Then, one week later, transient middle-cerebral artery occlusion (MCAO) was applied to induce cerebral ischemia. To label dividing cells in brain, the rats received injections with bromodeoxyuridine (BrdU) [46]. Neurogenesis and spine density were analyzed at day-1 and day-5 post MCAO. First of all, in intact rats, treatments with SERMs and estrogen resulted in a robust induction of neurogenesis in the ipsilateral subventricular zone (SVZ). Moreover, following ischemia treated with estrogen and raloxifene, neurogenesis in the ipsilateral SVZ was significantly higher than in placebo-treated rats [46]. Tamoxifen did not have this regenerating effect. Nonetheless, both SERMs, as well as estrogen, significantly attenuated spine density loss in the cortex at day-5 post ischemia. Thus, SERMs can induce significant repair processes in ischemic brain. Obviously, these results and those of the preceding sections should have a major effect on our thinking regarding the completeness whereby SERMs can counteract brain disease and brain injury, meaning, raloxifene may serve—not only to protect from neural damage—but also serve to repair brain damage.

## 6. Summary and Conclusions

At this day and time, a field of five pharmaceutical entities—fucoidan (a brown seaweed product), raloxifene (an SERM), SMM-189 (a CB-2 receptor ligand), pifithrin (a P53 modulator), and ligands (for TSPO) present comparable beneficial effects regarding healing from neurodegeneration, namely, restoration of structure and function by more than 90%, according to the standards of the studies in question (this could be seen in treatments of TBI) [1,26,28,29,119]. This is discussed in Section 2. Raloxifene in the context of other advances in treatments of neurodegeneration. Raloxifene, however, presents some advantages over the other compounds. In particular, already for two decades it has been used in medical practice for various chronic ailments in humans. In clinical trials, it also showed favorable effects regarding mild cognitive impairments in humans. Anecdotal evidence suggests that a substantial number of cases of Alzheimer’s disease may be prevented (indicated in the schematic diagram of Figure 4), and occasionally, even ameliorated. At whole CNS levels, raloxifene showed preventive effects regarding Alzheimer’s disease, i.e., mild cognitive impairments could be attenuated and, thus, progress toward full-blown Alzheimer’s dementia arrested.

Potentially more than with Alzheimer’s disease, raloxifene was quite effective in animal models of Parkinson’s disease (see scheme of Figure 4). In other animal models, it was indicated that raloxifene had curative effects regarding stroke and TBI, including regeneration of neurons (Figure 4). Importantly, as found with TBI, as late as 48 h after application of brain damage initiation, raloxifene appears to be able to achieve full brain repair, including restoration of normal behavior [1]. Regarding cell types, raloxifene protects neurons from cell death, prevents glial activation, ameliorates myelin damage, and maintains health of endothelial cells. Furthermore, in cell culture studies, raloxifene showed healing effects regarding multiple sclerosis (MS) and amyotrophic lateral sclerosis (ALS).

Regarding mechanisms, raloxifene is able to favorably modulate the adverse biological signals typical of brain disease and brain injury. These signals that can be modulated by raloxifene relate to neuronal activity, neurotransmitters, and their receptors, plasticity, inflammation, oxidative stress, nitric oxide, calcium homeostasis, behavioral impairments, cell death, etc. (Figure 4). These effects of raloxifene can be attained, on the one hand, by way of changes in gene expression of the relevant proteins, for example via its binding to the cell nuclear estrogen receptors ERα and ERβ, i.e., genomic pathways, and, on the other hand, by acting on the nongenomic pathway presented by the GPER plasma membrane estrogen receptor, and modulating its effects, receptors other than estrogen receptors (as reported for the CB-2 receptor), as well as direct interference with various other nongenomic pathways, for example, degrading Abeta, reducing oxidative stress, supporting homeostasis of glucose metabolism, modulating Ca^++^ balance, attenuating adverse mitochondrial activity, including prevention of programmed cell death, and modulation of intracellular cholesterol levels (Figure 3). Thus, raloxifene not only appears efficacious, but its two decade long use is also reflected by a wide spectrum of parameters that have been assayed to substantiate raloxifene’s effectiveness for treatments of brain damage due to injury and disease. The range of this spectrum of parameters is from molecular biological mechanism, cell viability, tissue regeneration, and behavioral assays to clinical trials. This not only provides for understanding mechanisms in association with neurodegeneration regarding raloxifene, but this may also be relevant for other promising agents, and potentially give directions for further drug development of other novel compounds to treat brain damage due to injury and disease.

In this review, attention was also given to the relatively new insights that Alzheimer’s disease can be caused by infections, bacterial as well as viral, and the most recent findings that raloxifene can counteract at least some bacterial and viral infections (see Figure 4). It was suggested that, foreknowledge, in which patients would show positive outcomes, would help to apply raloxifene prophylactically [100] (or prophylactic application of other antiviral and antibacterial agents, for that matter). It may be that, if one knows which infection a person has, and how the person performs on a cognitive test, one may be able to predict whether the person in the future would receive a diagnosis for Alzheimer’s disease if not treated prophylactically. As raloxifene may provide such a prophylactic treatment, this review pays attention to the molecular biological pathways that may be utilized by raloxifene to attenuate neurodegeneration underlying chronic progressing of brain damage due to brain injury and brain disease. Compounds, in addition to raloxifene, that may be potentially efficacious regarding treatment of brain damage caused by disease and injury, on the one hand, may work partly via different functional pathways, but on the second hand, may also share functional pathways (for example see, [18,19,20,21,22,23,24,25,26,27,28,29]). Thus, while each compound may have the capacity to be sufficient by itself, they may also complement each other’s effects.

In short, the potential of SERMs, including raloxifene, as well as other compounds, present hopeful developments for treatments of brain injury and brain disease. Thus, further studies of the beneficial effects of raloxifene regarding brain damage are warranted. A relatively simple clinical trial would be the application of raloxifene as treatment for TBI. In consideration for future human studies of raloxifene, to gain further insights regarding effects on neurodegeneration, cognitive impairment, and dementia, dosage of 120 mg/kg of raloxifene (or higher, as up to 600 mg/kg) appear to be optimal. Since the SERM raloxifene presents itself as a robust anti-neurodegeneration drug, for various brain injuries and brain diseases (Figure 4), it could be taken as a template standard for the design and testing of relative effectiveness of other novel anti-neurodegeneration medication. This approach may give guidance to what should be paid attention to when designing, developing, and testing compounds for characteristics important for attenuation of brain damage due to disease or injury.

## References

[1] Honig M.G., Del Mar N.A., Henderson D.L., Ragsdale T.D., Doty J.B., Driver J.H., Li C., Fortugno A.P., Mitchell W.M., Perry A.M. (2019). Amelioration of visual deficits and visual system pathology after mild TBI via the cannabinoid Type-2 receptor inverse agonism of raloxifene. Exp. Neurol..

[2] Gizzo S., Saccardi C., Patrelli T.S., Berretta R., Capobianco G., Di Gangi S., Vacilotto A., Bertocco A., Noventa M., Ancona E. (2013). Update on raloxifene: Mechanism of action, clinical efficacy, adverse effects, and contraindications. Obstet Gynecol. Surv..

[3] Lee W.-L., Cheng M.-H., Tarng D.-C., Yang W.-C., Lee F.-K., Wang P.-H. (2013). The benefits of estrogen or selective estrogen receptor modulator on kidney and its related disease—Chronic kidney disease—Mineral and bone disorder: Osteoporosis. J. Chin. Med. Assoc..

[4] Nickelsen T., Lufkin E.G., Riggs B.L., Cox D.A., Crook T.H. (1999). Raloxifene hydrochloride, a selective estrogen receptor modulator: Safety assessment of effects on cognitive function and mood in postmenopausal women. Psychoneuroendocrinology.

[5] Yang Z.D., Yu J., Zhang Q. (2013). Effects of raloxifene on cognition, mental health, sleep and sexual function in menopausal women: A systematic review of randomized controlled trials. Maturitas.

[6] Prokai-Tatrai K., Prokai L. (2019). A Novel Prodrug Approach for Central Nervous System-Selective Estrogen Therapy. Molecules.

[7] Uebelhart B., Herrmann F., Rizzoli R. (2009). Effects of the SERM raloxifene on calcium and phosphate metabolism in healthy middle-aged men. Clin. Cases Miner. Bone Metab..

[8] Smith M.R., Fallon M.A., Lee H., Finkelstein J.S. (2004). Raloxifene to prevent gonadotropin-releasing hormone agonist-induced bone loss in men with prostate cancer: A randomized controlled trial. J. Clin. Endocrinol. Metab..

[9] Dimaraki E.V., Symons K.V., Barkan A.L. (2004). Raloxifene decreases serum IGF-I in male patients with active acromegaly. Eur. J. Endocrinol..

[10] Neubauer B.L., Best K.L., Clemens J.A., Gates C.A., Goode R.L., Jones C.D., Laughlin M.E., Shaar C.J., Toomey R.E., Hoover D.M. (1993). Endocrine and antiprostatic effects of raloxifene (LY156758) in the male rat. Prostate.

[11] Yang Z., He X., Zhang Y. (2007). The determination of raloxifene in rat tissue using HPLC. Biomed. Chromatogr..

[12] Halbreich U., Kahn L.S. (2000). Selective oestrogen receptor modulators - current and future brain and behaviour applications. Expert Opin. Pharmacother..

[13] Littleton-Kearney M.T., Ostrowski N.L., Cox D.A., Rossberg M.I., Hurn P.D. (2002). Selective estrogen receptor modulators: Tissue actions and potential for CNS protection. CNS Drug Rev..

[14] Arevalo M.-A., Santos-Galindo M., Lagunas N., Azcoitia I., Garcia-Segura L.M. (2011). Selective estrogen receptor modulators as brain therapeutic agents. J. Mol. Endocrinol..

[15] Dhandapani K.M., Brann D.W. (2002). Protective effects of estrogen and selective estrogen receptor modulators in the brain. Biol. Reprod..

[16] Barreto G.E., Santos-Galindo M., Garcia-Segura L.M. (2014). Selective estrogen receptor modulators regulate reactive microglia after penetrating brain injury. Front. Aging Neurosci..

[17] Shehadeh M., Palzur E., Apel L., Soustiel J.F. (2019). Reduction of Traumatic Brain Damage by Tspo Ligand Etifoxine. Int. J. Mol. Sci..

[18] Gonzales-Portillo B., Lippert T., Nguyen H., Lee J.-Y., Borlongan C.V. (2019). Hyperbaric oxygen therapy: A new look on treating stroke and traumatic brain injury. Brain Circ..

[19] Weaver L.K., Churchill S., Wilson S.H., Hebert D., Deru K., Lindblad A.S. (2019). A composite outcome for mild traumatic brain injury in trials of hyperbaric oxygen. Undersea Hyperb. Med..

[20] Harch P.G., Andrews S.R., Rowe C.J., Lischka J.R., Townsend M.H., Yu Q., Mercante D.E. (2020). Hyperbaric oxygen therapy for mild traumatic brain injury persistent postconcussion syndrome: A randomized controlled trial. Med. Gas Res..

[21] Culmsee C., Zhu X., Yu Q.-S., Chan S.L., Camandola S., Guo Z., Greig N.H., Mattson M.P. (2001). A synthetic inhibitor of p53 protects neurons against death induced by ischemic and excitotoxic insults, and amyloid β-peptide. J. Neurochem..

[22] Arai M., Imamura O., Kondoh N., Dateki M., Takishima K. (2019). Neuronal Ca 2+ -dependent activator protein 1 (NCDAP1) induces neuronal cell death by activating p53 pathway following traumatic brain injury. J. Neurochem..

[23] Presley C., Abidi A., Suryawanshi S., Mustafa S., Meibohm B., Moore B.M. (2015). Preclinical evaluation of SMM-189, a cannabinoid receptor 2-specific inverse agonist. Pharmacol. Res. Perspect..

[24] Bu W., Ren H., Deng Y., Del Mar N., Guley N.M., Moore B.M., Honig M.G., Reiner A. (2016). Mild Traumatic Brain Injury Produces Neuron Loss That Can Be Rescued by Modulating Microglial Activation Using a CB2 Receptor Inverse Agonist. Front. Neurosci..

[25] Guley N.M., Del Mar N.A., Ragsdale T., Li C., Perry A.M., Moore B.M., Honig M.G., Reiner A. (2019). Amelioration of visual deficits and visual system pathology after mild TBI with the cannabinoid type-2 receptor inverse agonist SMM-189. Exp. Eye Res..

[26] Yu Y., Li L., Nguyen D.T., Mustafa S.M., Moore B.M., Jiang J. (2020). Inverse Agonism of Cannabinoid Receptor Type 2 Confers Anti-inflammatory and Neuroprotective Effects Following Status Epileptics. Mol. Neurobiol..

[27] Veenman L., Vainshtein A., Gavish M. (2015). TSPO as a target for treatments of diseases, including neuropathological disorders. Cell Death Dis..

[28] Dimitrova-Shumkovska J., Krstanoski L., Veenman L. (2020). Diagnostic and Therapeutic Potential of TSPO Studies Regarding Neurodegenerative Diseases, Psychiatric Disorders, Alcohol Use Disorders, Traumatic Brain Injury, and Stroke: An Update. Cells.

[29] Dimitrova-Shumkovska J., Krstanoski L., Veenman L. (2020). Potential Beneficial Actions of Fucoidan in Brain and Liver Injury, Disease, and Intoxication—Potential Implication of Sirtuins. Mar. Drugs.

[30] Pastuhov S.I., Hisamoto N., Matsumoto K. (2015). MAP kinase cascades regulating axon regeneration in C. elegans. Proc. Jpn. Acad. Ser. B Phys. Biol. Sci..

[31] Liddelow S.A., Barres B.A. (2017). Reactive Astrocytes: Production, Function, and Therapeutic Potential. Immunity.

[32] Shabab T., Khanabdali R., Moghadamtousi S.Z., Kadir H.A., Mohan G. (2017). Neuroinflammation pathways: A general review. Int. J. Neurosci..

[33] Güttinger H.R., Herth G., Stocker S., Kafitz K.W., Kallenbach M. (1993). Antioestrogen inhibits myelination in brains of juvenile Zebra finches. Neuroreport.

[34] Zhang L.N., Li M.J., Shang Y.H., Zhao F.F., Huang H.C., Lao F.X. (2020). Independent and Correlated Role of Apolipoprotein E ɛ4 Genotype and Herpes Simplex Virus Type 1 in Alzheimer’s Disease. J. Alzheimers Dis..

[35] Ciriza I., Carrero P., Azcoitia I., Lundeen S.G., Garcia-Segura L.M. (2004). Selective estrogen receptor modulators protect hippocampal neurons from kainic acid excitotoxicity: Differences with the effect of estradiol. J. Neurobiol..

[36] González-Burgos I., Rivera-Cervantes M.C., Velázquez-Zamora D.A., Feria-Velasco A., Garcia-Segura L.M. (2012). Selective estrogen receptor modulators regulate dendritic spine plasticity in the hippocampus of male rats. Neural Plast..

[37] Velázquez-Zamora D.A., Garcia-Segura L.M., González-Burgos I. (2012). Effects of selective estrogen receptor modulators on allocentric working memory performance and on dendritic spines in medial prefrontal cortex pyramidal neurons of ovariectomized rats. Horm. Behav..

[38] Lagunas N., Calmarza-Font I., Grassi D., Garcia-Segura L.M. (2011). Estrogen receptor ligands counteract cognitive deficits caused by androgen deprivation in male rats. Horm. Behav..

[39] Yaffe K., Krueger K., Cummings S.R., Blackwell T., Henderson V.W., Sarkar S., Ensrud K., Grady D. (2005). Effect of raloxifene on prevention of dementia and cognitive impairment in older women: The Multiple Outcomes of Raloxifene Evaluation (MORE) randomized trial. Am. J. Psychiatry.

[40] Yaffe K., Blackwell T., Whitmer R.A., Krueger K., Barrett-Connor E. (2006). Glycosylated hemoglobin level and development of mild cognitive impairment or dementia in older women. J. Nutr. Health Aging.

[41] Jacobsen D.E., Samson M.M., Emmelot-Vonk M.H., Verhaar H.J. (2010). Raloxifene improves verbal memory in late postmenopausal women: A randomized, double-blind, placebo-controlled trial. Menopause.

[42] Schroeder A., Hudson M.R., Du X., Wu Y.W.C., Nakamura J., van den Buuse M., Jones N.C., Hill R.A. (2017). Estradiol and raloxifene modulate hippocampal gamma oscillations during a spatial memory task. Psychoneuroendocrinology.

[43] Lampe P.D., Lau A.F. (2000). Regulation of gap junctions by phosphorylation of connexins. Arch. Biochem. Biophys..

[44] Lampe P.D., Lau A.F. (2004). The effects of connexin phosphorylation on gap junctional communication. Int. J. Biochem. Cell Biol..

[45] Dahm L., Klugmann F., Gonzalez-Algaba A., Reuss B. (2010). Tamoxifen and raloxifene modulate gap junction coupling during early phases of retinoic acid-dependent neuronal differentiation of NTera2/D1 cells. Cell Biol. Toxicol..

[46] Khan M.M., Wakade C., de Sevilla L., Brann D.W. (2015). Selective estrogen receptor modulators (SERMs) enhance neurogenesis and spine density following focal cerebral ischemia. J. Steroid Biochem. Mol. Biol..

[47] Uryash A., Flores V., Adams J.A., Allen P.D., Lopez J.R. (2020). Memory and Learning Deficits Are Associated With Ca2+ Dyshomeostasis in Normal Aging. Front. Aging Neurosci..

[48] Zhou X., Yang Z., Han L., Li X., Feng M., Zhang T., Luo H., Zhu L., Zhang J., Zhang Q. (2015). Raloxifene neutralizes the adverse effects of glutamate on cultured neurons by regulation of calcium oscillations. Mol. Med. Rep..

[49] Huang Y.L., Lai B., Zheng P., Zhu Y.C., Yao T. (2007). Raloxifene acutely reduces glutamate-induced intracellular calcium increase in cultured rat cortical neurons via inhibition of high-voltage-activated calcium current. Neuroscience.

[50] Barreto G., Santos-Galindo M., Diz-Chaves Y., Pernía O., Carrero P., Azcoitia I., Garcia-Segura L.M. (2009). Selective estrogen receptor modulators decrease reactive astrogliosis in the injured brain: Effects of aging and prolonged depletion of ovarian hormones. Endocrinology.

[51] Pekny M., Pekna M. (2014). Astrocyte reactivity and reactive astrogliosis: Costs and benefits. Physiol. Rev..

[52] Escartin C., Guillemaud O., Carrillo-de Sauvage M.A. (2019). Questions and (some) answers on reactive astrocytes. Glia.

[53] Li T., Chen X., Zhang C., Zhang Y., Yao W. (2019). An update on reactive astrocytes in chronic pain. J. Neuroinflammation.

[54] Li K., Li J., Zheng J., Qin S. (2019). Reactive Astrocytes in Neurodegenerative Diseases. Aging Dis..

[55] Cerciat M., Unkila M., Garcia-Segura L.M., Arevalo M.A. (2010). Selective estrogen receptor modulators decrease the production of interleukin-6 and interferon-gamma-inducible protein-10 by astrocytes exposed to inflammatory challenge in vitro. Glia.

[56] Arevalo M.A., Diz-Chaves Y., Santos-Galindo M., Bellini M.J., Garcia-Segura L.M. (2011). Selective oestrogen receptor modulators decrease the inflammatory response of glial cells. J. Neuroendocr..

[57] Vesga-Jiménez D.J., Hidalgo-Lanussa O., Baez-Jurado E., Echeverria V., Ashraf G.M., Sahebkar A., Barreto G.E. (2019). Raloxifene attenuates oxidative stress and preserves mitochondrial function in astrocytic cells upon glucose deprivation. J. Cell. Physiol..

[58] Del Rio-Hortega P. (2012). Studies on neuroglia: Glia with very few processes (oligodendroglia) by PA-o del RA-o-Hortega. 1921. Clin. Neuropathol..

[59] Tronel C., Largeau B., Santiago Ribeiro M.J., Guilloteau D., Dupont A.C., Arlicot N. (2017). Molecular Targets for PET Imaging of Activated Microglia: The Current Situation and Future Expectations. Int. J. Mol. Sci..

[60] Arulsamy A., Teng J., Colton H., Corrigan F., Collins-Praino L. (2018). Evaluation of early chronic functional outcomes and their relationship to pre-frontal cortex and hippocampal pathology following moderate-severe traumatic brain injury. Behav. Brain Res..

[61] Skaper S.D., Facci L., Zusso M., Giusti P. (2018). An Inflammation-Centric View of Neurological Disease: Beyond the Neuron. Front. Cell. Neurosci..

[62] Colonna M., Butovsky O. (2017). Microglia Function in the Central Nervous System during Health and Neurodegeneration. Annu. Rev. Immunol..

[63] García-Revilla J., Alonso-Bellido I.M., Burguillos M.A., Herrera A.J., Espinosa-Oliva A.M., Ruiz R., Cruz-Hernández L., García-Domínguez I., Roca-Ceballos M.A., Santiago M. (2019). Reformulating Pro-Oxidant Microglia in Neurodegeneration. J. Clin. Med..

[64] Paolicelli R.C., Bolasc G., Pagani F., Maggi L., Scianni M., Panzanelli P., Giustetto M., Ferreira T.A., Guiducci E., Dumas L. (2011). Synaptic pruning by microglia is necessary for normal brain development. Science.

[65] Cunningham C.L., Martinez-Cerdeno V., Noctor S.C. (2013). Microglia regulate the number of neural precursor cells in the developing cerebral cortex. J. Neurosci..

[66] Squarzoni P., Oller G., Hoeffel G., Pont-Lezica L., Rostaing P., Low D., Bessis A., Ginhoux F., Garel S. (2014). Microglia modulate wiring of the embryonic forebrain. Cell Rep..

[67] Suuronen T., Nuutinen T., Huuskonen J., Ojala J., Thornell A., Salminen A. (2005). Anti-inflammatory effect of selective estrogen receptor modulators (SERMs) in microglial cells. Inflamm. Res..

[68] Tapia-Gonzalez S., Carrero P., Pernia O., Garcia-Segura L.M., Diz-Chaves Y. (2008). Selective oestrogen receptor (ER) modulators reduce microglia reactivity in vivo after peripheral inflammation: Potential role of microglial ERs. J. Endocrinol..

[69] Ishihara Y., Itoh K., Ishida A., Yamazaki T. (2015). Selective estrogen-receptor modulators suppress microglial activation and neuronal cell death via an estrogen receptor-dependent pathway. J. Steroid Biochem. Mol. Biol..

[70] Hu X., Qin X. (2013). Lentivirus-mediated estrogen receptor α overexpression in the central nervous system ameliorates experimental autoimmune encephalomyelitis in mice. Int. J. Mol. Med..

[71] Deanfield J.E., Halcox J.P., Rabelink T.J. (2007). Endothelial function and dysfunction. Circulation.

[72] Oge A., Sezer E.D., Ozgönül M., Bayraktar F., Sözmen E.Y. (2003). The effects of estrogen and raloxifene treatment on the antioxidant enzymes and nitrite-nitrate levels in brain cortex of ovariectomized rats. Neurosci. Lett..

[73] Razmara A., Sunday L., Stirone C., Wang X.B., Krause D.N., Duckles S.P., Procaccio V. (2008). Mitochondrial effects of estrogen are mediated by estrogen receptor α in brain endothelial cells. J. Pharmacol. Exp. Ther..

[74] Allais G., Chiarle G., Sinigaglia S., Airola G., Schiapparelli P., Benedetto C. (2020). Gender-related differences in migraine. Neurol. Sci..

[75] Ozgönül M., Oge A., Sezer E.D., Bayraktar F., Sözmen E.Y. (2003). The effects of estrogen and raloxifene treatment on antioxidant enzymes in brain and liver of ovarectomized female rats. Endocr. Res..

[76] Konyalioglu S., Durmaz G., Yalcin A. (2007). The potential antioxidant effect of raloxifene treatment: A study on heart, liver and brain cortex of ovariectomized female rats. Cell Biochem. Funct..

[77] Yazğan Y., Nazıroğlu M. (2017). Ovariectomy-Induced Mitochondrial Oxidative Stress, Apoptosis, and Calcium Ion Influx Through TRPA1, TRPM2, and TRPV1 Are Prevented by 17β-Estradiol, Tamoxifen, and Raloxifene in the Hippocampus and Dorsal Root Ganglion of Rats. Mol. Neurobiol..

[78] Bai J., Qi Q.R., Li Y., Day R., Makhoul J., Magness R.R., Chen D.B. (2020). Estrogen Receptors and Estrogen-Induced Uterine Vasodilation in Pregnancy. Int. J. Mol. Sci..

[79] Teoh J.-P., Li X., Simoncini T., Zhu D., Fu X. (2020). Estrogen-Mediated Gaseous Signaling Molecules in Cardiovascular Disease. Trends Endocrinol. Metab..

[80] Carroll J.S. (2016). Mechanisms of oestrogen receptor (ER) gene regulation in breast cancer. Eur. J. Endocrinol..

[81] Kumar A., Foster T.C. (2020). G Protein-Coupled Estrogen Receptor: Rapid Effects on Hippocampal-Dependent Spatial Memory and Synaptic Plasticity. Front. Endocrinol..

[82] Wu X., Glinn M.A., Ostrowski N.L., Su Y., Ni B., Cole H.W., Bryant H.U., Paul S.M. (1999). Raloxifene and estradiol benzoate both fully restore hippocampal choline acetyltransferase activity in ovariectomized rats. Brain Res..

[83] Cyr M., Morissette M., Landry M., Di Paolo T. (2001). Estrogenic activity of tamoxifen and raloxifene on rat brain AMPA receptors. Neuroreport.

[84] Yalcin A., Kanit L., Durmaz G., Sargin S., Terek C.H., Tanyolac B. (2005). Altered level of apurinic/apyrimidinic endonuclease/redox factor-1 (APE/REF-1) mRNA in the hippocampus of ovariectomized rats treated by raloxifene against kainic acid. Clin. Exp. Pharmacol. Physiol..

[85] Lee Y.R., Joo H.K., Jeon B.H. (2020). The Biological Role of Apurinic/Apyrimidinic Endonuclease1/Redox Factor-1 as a Therapeutic Target for Vascular Inflammation and as a Serologic Biomarker. Biomedicines.

[86] Yazğan B., Yazğan Y., Övey İ.S., Nazıroğlu M. (2016). Raloxifene and Tamoxifen Reduce PARP Activity, Cytokine and Oxidative Stress Levels in the Brain and Blood of Ovariectomized Rats. J. Mol. Neurosci..

[87] Herceg Z., Wang Z.Q. (2001). Functions of poly(ADP-ribose) polymerase (PARP) in DNA repair, genomic integrity and cell death. Mutat. Res..

[88] Rzemieniec J., Litwa E., Wnuk A., Lason W., Kajta M. (2018). Bazedoxifene and raloxifene protect neocortical neurons undergoing hypoxia via targeting ERα and PPAR-γ. Mol. Cell. Endocrinol..

[89] Bethea C.L., Mirkes S.J., Su A., Michelson D. (2002). Effects of oral estrogen, raloxifene and arzoxifene on gene expression in serotonin neurons of macaques. Psychoneuroendocrinology.

[90] Smith L.J., Henderson J.A., Abell C.W., Bethea C.L. (2004). Effects of ovarian steroids and raloxifene on proteins that synthesize, transport, and degrade serotonin in the raphe region of macaques. Neuropsychopharmacology.

[91] Sumner B.E., Grant K.E., Rosie R., Hegele-Hartung C., Fritzemeier K.H., Fink G. (2007). Raloxifene blocks estradiol induction of the serotonin transporter and 5-hydroxytryptamine2A receptor in female rat brain. Neurosci. Lett..

[92] Landry M., Lévesque D., Di Paolo T. (2002). Estrogenic properties of raloxifene, but not tamoxifen, on D2 and D3 dopamine receptors in the rat forebrain. Neuroendocrinology.

[93] Le Saux M., Di Paolo T. (2005). Chronic estrogenic drug treatment increases preproenkephalin mRNA levels in the rat striatum and nucleus accumbens. Psychoneuroendocrinology.

[94] Zhou F., Dong H., Liu Y., Yan L., Sun C., Hao P., Liu Y., Zhai J., Liu Y. (2018). Raloxifene, a promising estrogen replacement, limits TDP-25 cell death by enhancing autophagy and suppressing apoptosis. Brain Res. Bull..

[95] Xu S., Yu S., Dong D., Lee L.T.O. (2019). G Protein-Coupled Estrogen Receptor: A Potential Therapeutic Target in Cancer. Front. Endocrinol..

[96] Salort G., Álvaro-Bartolomé M., García-Sevilla J.A. (2017). Regulation of cannabinoid CB2 receptor constitutive activity in vivo: Repeated treatments with inverse agonists reverse the acute activation of JNK and associated apoptotic signaling in mouse brain. Psychopharmacology.

[97] Du X., Hill R.A. (2016). The Potential of Gonadal Hormone Signalling Pathways as Therapeutics for Dementia. J. Mol. Neurosci..

[98] Zec R.F., Trivedi M.A. (2002). The effects of estrogen replacement therapy on neuropsychological functioning in postmenopausal women with and without dementia: A critical and theoretical review. Neuropsychol. Rev..

[99] Ritchie K., Touchon J. (2000). Mild cognitive impairment: Conceptual basis and current nosological status. Lancet.

[100] Cummings J.L. (2005). Searching for methods to detect, prevent, and treat Alzheimer’s disease. Am. J. Psychiatry.

[101] Henderson V.W., Ala T., Sainani K.L., Bernstein A.L., Stephenson B.S., Rosen A.C., Farlow M.R. (2015). Raloxifene for women with Alzheimer disease: A randomized controlled pilot trial. Neurology.

[102] Pinsky P.F., Miller E.A., Heckman-Stoddard B.M., Minasian L. (2020). Breast Cancer Characteristics and Survival among Users versus Nonusers of Raloxifene. Cancer Prev. Res..

[103] Peri A., Benvenuti S., Luciani P., Deledda C., Cellai I. (2011). Membrane cholesterol as a mediator of the neuroprotective effects of estrogens. Neuroscience.

[104] Callier S., Morissette M., Grandbois M., Pélaprat D., Di Paolo T. (2001). Neuroprotective properties of 17β-estradiol, progesterone, and raloxifene in MPTPC57Bl/6 mice. Synapse.

[105] Liu Z., Wang Y., Qin W., Chen D., Feng Y., Su H., Shao W., Zhou B., Bu X. (2019). Raloxifene alleviates amyloid-β-induced cytotoxicity in HT22 neuronal cells via inhibiting oligomeric and fibrillar species formation. J. Biochem. Mol. Toxicol..

[106] Neele S.J., Rombouts S.A., Bierlaagh M.A., Barkhof F., Scheltens P., Netelenbos J.C. (2001). Raloxifene affects brain activation patterns in postmenopausal women during visual encoding. J. Clin. Endocrinol. Metab..

[107] Goekoop R., Barkhof F., Duschek E.J., Netelenbos C., Knol D.L., Scheltens P., Rombouts S.A. (2006). Raloxifene treatment enhances brain activation during recognition of familiar items: A pharmacological fmri study in healthy elderly males. Neuropsychopharmacology.

[108] Block J. (2019). Alzheimer’s disease might depend on enabling pathogens which do not necessarily cross the blood-brain barrier. Med. Hypotheses.

[109] Shah A.F., Morris J.A., Wray M. (2020). Pathogenesis of Alzheimer’s disease: Multiple interacting causes against which amyloid precursor protein protects. Med. Hypotheses.

[110] Eyre N.S., Kirby E.N., Anfiteatro D.R., Bracho G., Russo A.G., White P.A., Aloia A.L., Beard M.R. (2020). Identification of Estrogen Receptor Modulators as Inhibitors of Flavivirus Infection. Antimicrob. Agents Chemother..

[111] KalantarMotamedi Y., Eastman R.T., Guha R., Bender A. (2018). A systematic and prospectively validated approach for identifying synergistic drug combinations against malaria. Malar. J..

[112] Sawada H., Shimohama S. (2003). Estrogens and parkinson disease: Novel approach for neuroprotection. Endocrine.

[113] Ramirez A.D., Liu X., Menniti F.S. (2003). Repeated estradiol treatment prevents MPTP-induced dopamine depletion in male mice. Neuroendocrinology.

[114] D’Astous M., Morissette M., Tanguay B., Callier S., Di Paolo T. (2003). Dehydroepiandrosterone (DHEA) such as 17β-estradiol prevents MPTP-induced dopamine depletion in mice. Synapse.

[115] Bourque M., Morissette M., Di Paolo T. (2014). Raloxifene activates G protein-coupled estrogen receptor 1/Akt signaling to protect dopamine neurons in 1-methyl-4-phenyl-1,2,3,6-tetrahydropyridine mice. Neurobiol. Aging.

[116] Baraka A.M., Korish A.A., Soliman G.A., Kamal H. (2011). The possible role of estrogen and selective estrogen receptor modulators in a rat model of Parkinson’s disease. Life Sci..

[117] Zhang D., Yang S., Yang C., Jin G., Zhen X. (2008). Estrogen regulates responses of dopamine neurons in the ventral tegmental area to cocaine. Psychopharmacology.

[118] Kokiko O.N., Murashov A.K., Hoane M.R. (2006). Administration of raloxifene reduces sensorimotor and working memory deficits following traumatic brain injury. Behav. Brain Res..

[119] Yang L.-Y., Greig N.H., Tweedie D., Jung Y.J., Chiang Y.-H., Hoffer B.J., Miller J.P., Chang K.-H., Wang J.-Y. (2020). The p53 inactivators pifithrin-μ and pifithrin-α mitigate TBI-induced neuronal damage through regulation of oxidative stress, neuroinflammation, autophagy and mitophagy. Exp. Neurol..

